# Meta-analysis of human and mouse ALS astrocytes reveals multi-omic signatures of inflammatory reactive states

**DOI:** 10.1101/gr.275939.121

**Published:** 2022-01

**Authors:** Oliver J. Ziff, Benjamin E. Clarke, Doaa M. Taha, Hamish Crerar, Nicholas M. Luscombe, Rickie Patani

**Affiliations:** 1 Department of Neuromuscular Diseases, Queen Square Institute of Neurology, University College London, London WC1N 3BG, United Kingdom;; 2 The Francis Crick Institute, London NW1 1AT, United Kingdom;; 3 National Hospital for Neurology and Neurosurgery, University College London NHS Foundation Trust, London WC1N 3BG, United Kingdom;; 4 Department of Zoology, Faculty of Science, Alexandria University, Alexandria 21511, Egypt;; 5 UCL Genetics Institute, University College London, London WC1E 6BT, United Kingdom;; 6 Okinawa Institute of Science and Technology Graduate University, Okinawa 904-0495, Japan

## Abstract

Astrocytes contribute to motor neuron death in amyotrophic lateral sclerosis (ALS), but whether they adopt deleterious features consistent with inflammatory reactive states remains incompletely resolved. To identify inflammatory reactive features in ALS human induced pluripotent stem cell (hiPSC)–derived astrocytes, we examined transcriptomics, proteomics, and glutamate uptake in *VCP*-mutant astrocytes. We complemented this by examining other ALS mutations and models using a systematic meta-analysis of all publicly-available ALS astrocyte sequencing data, which included hiPSC-derived astrocytes carrying *SOD1*, *C9orf72*, and *FUS* gene mutations as well as mouse ALS astrocyte models with *SOD1^G93A^* mutation, *Tardbp* deletion, and *Tmem259* (also known as membralin) deletion. ALS astrocytes were characterized by up-regulation of genes involved in the extracellular matrix, endoplasmic reticulum stress, and the immune response and down-regulation of synaptic integrity, glutamate uptake, and other neuronal support processes. We identify activation of the TGFB, Wnt, and hypoxia signaling pathways in both hiPSC and mouse ALS astrocytes. ALS changes positively correlate with TNF, IL1A, and complement pathway component C1q-treated inflammatory reactive astrocytes, with significant overlap of differentially expressed genes. By contrasting ALS changes with models of protective reactive astrocytes, including middle cerebral artery occlusion and spinal cord injury, we uncover a cluster of genes changing in opposing directions, which may represent down-regulated homeostatic genes and up-regulated deleterious genes in ALS astrocytes. These observations indicate that ALS astrocytes augment inflammatory processes while concomitantly suppressing neuronal supporting mechanisms, thus resembling inflammatory reactive states and offering potential therapeutic targets.

Amyotrophic lateral sclerosis (ALS) is characterized by the selective degeneration of motor neurons and the presence of *TARDBP* pathology in >97% of cases (Neumann et al. 2006; Rohan et al. 2014; Al-Chalabi et al. 2017). Mutations in approximately 30 genes are causative for ALS, including *C9orf72*, *SOD1*, *FUS*, *TARDBP*, and *VCP* (Johnson et al. 2010; Al-Chalabi et al. 2017). Astrocytes have been heavily implicated in the pathology of ALS, through both gain of toxic factors and loss of homeostatic functions (Rothstein et al. 1990; Nagai et al. 2007; Yamanaka et al. 2008; Ferraiuolo et al. 2011; Meyer et al. 2014; Tyzack et al. 2017). Among these mechanisms, impaired astrocytic glutamate uptake leads to excitotoxicity and has been suggested to play an important role in motor neuron hyperexcitability and death in ALS (Rothstein et al. 1990, 1992; Guo et al. 2003; Van Den Bosch et al. 2006; Dafinca et al. 2020). The primary glutamate transporter, *SLC1A2* (also known as *EAAT2*) (responsible for ∼90% of CNS glutamate uptake) (Tanaka et al. 1997), has been shown to be markedly reduced in the spinal cord of ALS patients (Lin et al. 1998; Sasaki et al. 2000). Recently, the endoplasmic reticulum (ER) membrane protein, transmembrane protein 259 (*Tmem259*, also known as membralin), was shown to maintain expression of *Slc1a2*, and astrocyte-specific membralin deletion led to the accumulation of extracellular glutamate, resulting in motor neuron death (Jiang et al. 2019).

In response to a wide variety of stimuli, astrocytes can markedly change their gene expression, morphology, and function in a process termed “reactivity.” Astrocytes adopt unique reactive states depending on the context-specific stimuli that they encounter, which can have detrimental or protective effects on neurons (Anderson et al. 2016; Liddelow et al. 2017; Escartin et al. 2021). Incubating astrocytes in the proinflammatory factors, TNF, IL1A, and complement pathway component C1q (encoded by the *C1qa/b/c* genes and referred to as C1q hereafter), induces a deleterious reactive state, referred to as “A1” (Liddelow et al. 2017), with impairment of homeostatic functions including glutamate uptake in human induced pluripotent stem cell (hiPSC)–derived and mouse astrocytes (Liddelow et al. 2017; Barbar et al. 2020; Guttenplan et al. 2020; Leng et al. 2021). Triple knockout of *Tnf*, *Il1a*, and *C1qa* slowed disease progression in the *SOD1^G93A^* mouse model of ALS, directly implicating inducers of an A1-reactive state in ALS (Guttenplan et al. 2020). Furthermore, C3, which is a recognized marker of A1 astrocytes, has been identified in postmortem tissue of several neurodegenerative diseases including ALS (Liddelow et al. 2017; Guttenplan et al. 2020). However, it is currently unclear whether ALS astrocytes adopt deleterious features seen in astrocytes treated with TNF, IL1A, and C1q.

Using astrocyte and motor neuron coculture, we have previously shown that hiPSC-derived astrocytes carrying ALS-causing mutations in the *VCP* gene have reduced capacity to promote survival of *VCP*-mutant and control motor neurons (Hall et al. 2017). Furthermore, we recently reported that *VCP*-mutant astrocytes express the A1 marker, C3 and have a secretory profile suggestive of an inflammatory reactive state (Taha et al. 2021). Other ALS studies have also shown neuroprotective deficits in *SOD1* mutant hiPSC-astrocytes (Tyzack et al. 2017) and deleterious effects of conditioned media from *C9orf72* mutant hiPSC-astrocytes on neurons (Birger et al. 2019). Here, in the present study, we aimed to extend these previous findings by systematically evaluating (1) ALS reactive astrocyte molecular and functional features across different mutations and models, and (2) the degree to which ALS astrocytes resemble deleterious inflammatory astrocytes (induced by treatment of the healthy state with TNF, IL1A, and C1q) or neuroprotective astrocytes (induced by middle cerebral artery occlusion [MCAO] and spinal cord injury [SCI] models).

## Results

### Transcriptomic and functional validation of highly enriched hiPSC-astrocytes

**Figure 1.**
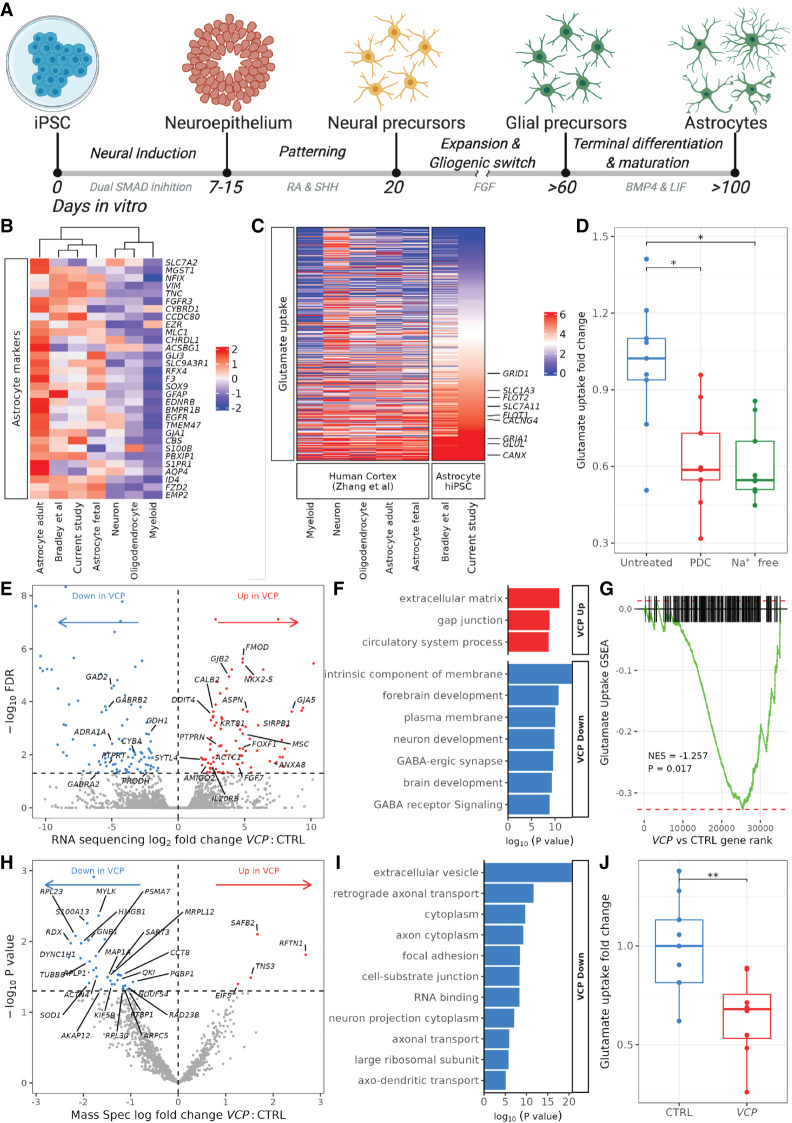
Characterization of control and *VCP*-mutant hiPSC-astrocyte cultures. (*A*) Schematic showing directed differentiation of hiPSC-astrocytes patterned to the ventral spinal cord. (*B*,*C*) Heatmaps showing the mean row-scaled variance stabilized gene counts of astrocyte-specific markers (*B*) and glutamate uptake genes (*C*) in our control hiPSC-astrocytes (current study), ventral spinal cord (VSC) regional hiPSC-astrocytes (Bradley et al. 2019), and human CNS tissue purified cell types (Zhang et al. 2016). For individual sample clustering, see Supplemental Figure S1. (*D*) Boxplot showing fold change in glutamate uptake of control hiPSC-astrocytes treated with 1 mM PDC or incubated in Na^+^-free media over untreated. N = 5 control lines each with one to three assays (experimental repeats) and each with one to three wells on the plate (technical replicates). A Wilcoxon test was used to compare groups (PDC vs. untreated, *P* = 0.012; Na^+^ free vs. untreated, *P* = 0.012). (*E*) Volcano plot showing log_2_ fold change in *VCP*-mutant versus control astrocyte differential gene expression from RNA sequencing. Genes with significantly (FDR < 0.05) increased expression are shown in red, and those decreased in expression are shown in blue. (*F*) Gene Ontology (GO) terms enriched in up-regulated (red) and down-regulated (blue) *VCP* versus control astrocyte differentially expressed genes from RNA sequencing. (*G*) Gene set enrichment plots showing changes in gene expression with *VCP* versus control astrocytes for the glutamate uptake gene set. (NES) Normalized enrichment score. (*H*) Volcano plot showing log fold change in *VCP*-mutant versus control astrocyte differential protein abundance from mass spectroscopy. (*I*) GO terms enriched in down-regulated differential proteins from mass spectroscopy in *VCP*-mutant versus control astrocytes (there were no terms associated with up-regulated proteins). (*J*) Fold change in glutamate uptake of control (blue) and *VCP*-mutant (red) astrocytes normalized to confluency. N = 5 control lines, four *VCP* lines, three technical replicates, one to four experimental repeats. To normalize for variation between the four experimental repeats, fold changes were calculated by dividing the mean of the three technical repeats over the control samples for each experimental repeat. Wilcoxon test: (**) *P* < 0.01.

We used our previously validated feeder-free and chemically defined hiPSC-derived astrocyte platform, which generates highly enriched (>90%) astrocyte monolayer cultures patterned to the ventral spinal cord (Fig. 1A; Hall et al. 2017; Tyzack et al. 2017; Kelley et al. 2018; Smethurst et al. 2020). These cells express astrocyte-specific markers and perform astrocytic functions, including cytosolic calcium responses to ATP and releasing cytokines in response to proinflammatory stimulation (Hall et al. 2017; Clarke et al. 2020; Thelin et al. 2020). To further verify the cellular identity of our cultures, we used deep high-throughput RNA sequencing from two lines derived from two patients with ALS-causing *VCP* gene mutations (p.R155C and p.R191Q) and two healthy control lines (Supplemental Table S1; Ziff et al. 2021). Principal component analysis and unsupervised hierarchical clustering showed that our control and *VCP*-mutant hiPSC-astrocyte samples cluster close to fetal primary human astrocytes and to other hiPSC-astrocyte data sets but are distinct from neurons, oligodendrocytes, and myeloid cells isolated from human brains (Supplemental Fig. S1A,B; Table 1; Zhang et al. 2016; Bradley et al. 2019). Clustering based only on known astrocyte-specific markers confirmed that our control hiPSC-astrocytes clustered with astrocyte samples, with high expression of *VIM*, *SOX9*, *FGFR3*, *MLC1*, *GFAP*, and *NFIX* (Fig. 1B). This also verified the ventral spinal cord specification of our cultures by clustering with region-specific hiPSC-astrocytes (Bradley et al. 2019). In addition, our control hiPSC-astrocytes highly expressed several genes known to be involved in glutamate uptake, including *CANX*, *GLUL*, *PTEN1*, *GRIA1*, *FLOT1*, *CACNG4*, *SLC7A11*, *FLOT2*, *SLC1A3*, and *GRID1* (Fig. 1C). We next used a functional assay to examine the capacity of our control hiPSC-astrocytes to perform glutamate uptake (Krencik et al. 2011; Barbar et al. 2020). We found that hiPSC-astrocytes performed Na^+^-dependent glutamate uptake, which was decreased by glutamate transporter inhibitor L-*trans*-pyrrolidine-2,4-dicarboxylic acid (PDC) or by incubation of cultures in Na^+^-free media (Fig. 1D). Taken together, this transcriptomic and functional characterization of our control hiPSC-astrocytes shows it to be a valuable in vitro model to explore molecular astrocytic functions in disease.

**Table 1.**
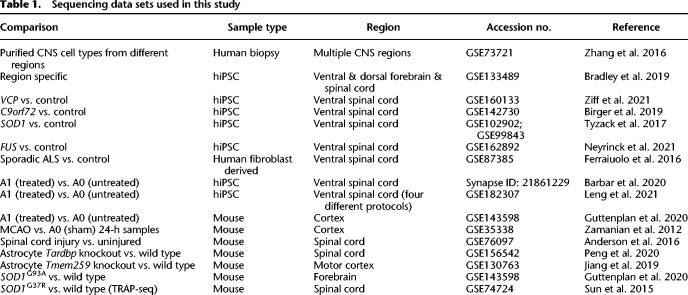
Sequencing data sets used in this study

### *VCP*-mutant hiPSC-derived astrocytes show transcriptomic and proteomic perturbations combined with functional deficits in glutamate uptake

We next sought to evaluate whether hiPSC-derived astrocytes from patients with ALS-causing *VCP* gene mutations showed disruption at the transcriptomic, proteomic, and functional levels (Hall et al. 2017). We first examined morphological parameters of astrocyte cultures using immunolabeling, which revealed a nonsignificant decrease in the size and an increase in the roundedness of *VCP*-mutant astrocytes as well as their nuclei compared with control astrocytes. However, we noted substantial heterogeneity between cell lines, consistent with reports of astrocyte diversity from other studies (Supplemental Fig. S2; Oberheim et al. 2012; Barbar et al. 2020). We next examined differences between *VCP*-mutant and control astrocyte gene expression patterns and identified 199 differentially expressed genes, with 91 up-regulated and 108 down-regulated in *VCP*-mutant astrocytes (FDR < 0.05) (Fig. 1E). Using Gene Ontology (GO) analysis, we found that up-regulated genes in *VCP*-mutant astrocytes were enriched in extracellular matrix (*FMOD*, *ASPN*, *OGN*, *ADAMTS8*) and cell junction (*GJB2*, *GJA5*, *CALB2*) processes, whereas down-regulated genes were overrepresented by GABA receptor signaling (*GABRA2*, *GABRB2*) and plasma membrane components (*FAM107A*, *SLC3A2*, *GAD2*), implicating aberrant astrocyte functions in the context of *VCP* mutations (Fig. 1F). Gene set enrichment analysis (GSEA) of glutamate uptake (gene set n = 403) showed significant down-regulation of glutamate uptake genes in *VCP*-mutant astrocytes (normalized enrichment score [NES] −1.3, enrichment *P* = 0.02), with decreases in *PTPRT*, *ADRA1A*, *GAD2*, *CDH1*, and *CDH10* (Fig. 1G). Using mass spectrometry, we identified 40 differentially expressed proteins, with four up-regulated and 36 down-regulated in *VCP*-mutant astrocytes (Fig. 1H; Supplemental Table S2). GO analysis revealed that down-regulated proteins were enriched in several processes, including axonal transport and focal adhesion (Fig. 1I). Furthermore, we confirmed a decrease in overall levels in 59% (26/44) of glutamate uptake–related proteins. We next determined whether these changes in glutamate uptake had a functional impact in *VCP*-mutant astrocytes and confirmed that *VCP*-mutant astrocytes displayed reduced glutamate uptake compared with that of control lines (Fig. 1J), indicating functional deficits in *VCP*-mutant hiPSC-derived astrocytes.

### Meta-analysis of ALS mutant astrocytes reveals common transcriptomic signatures with induced reactive astrocytes

To identify common changes across astrocytes carrying diverse ALS mutations, we reanalyzed published RNAseq datasets from ALS hiPSC-derived astrocytes carrying *C9orf72* (Birger et al. 2019)*, SOD1* (Tyzack et al. 2017), and *FUS* (Neyrinck et al. 2021) mutations (*C9orf72* data set: two mutant and two control lines; *SOD1*: three mutants and four controls; *FUS*: six mutants and six controls). *C9orf72* hiPSC-astrocytes were found to produce higher levels of radical oxygen species (ROS) and acquire a senescent phenotype, and conditioned media was deleterious to neurons (Birger et al. 2019). Meanwhile, *SOD1* and *FUS* hiPSC-astrocytes displayed transcriptional and proteomic reactivity related changes, such as STAT3 signaling (Tyzack et al. 2017; Neyrinck et al. 2021). In all three independent data sets, we found up-regulation of genes involved in extracellular matrix and cell adhesion, as well as down-regulation of neuronal- and synapse-related genes (Supplemental Fig. S3A–F). GSEA revealed that the glutamate uptake gene set was significantly decreased in expression with each mutation (*C9orf72* NES −1.7, enrichment *P* < 1 × 10^10^; *SOD1* NES −2.5, *P* < 1 × 10^10^; *FUS* NES −1.3, *P* = 0.01) (Supplemental Fig. S3G–I). To investigate whether ALS mutant astrocytes show differential expression within the same genes, we overlapped genes significantly changed in expression between *VCP, C9orf72*, *SOD1*, and *FUS* mutants (Supplemental Fig. S3J,K; Supplemental Table S3). Of note, in all four independent ALS mutant astrocyte data sets, gap junction protein beta 2 (*GJB2*) was significantly increased in expression, whereas *LRRC3B* (also known as *LRP15*) and MOB kinase activator 3B (*MOB3B*) were significantly decreased in expression—the latter of which itself shows ALS-associated loci (Li et al. 2021).

**Figure 2.**
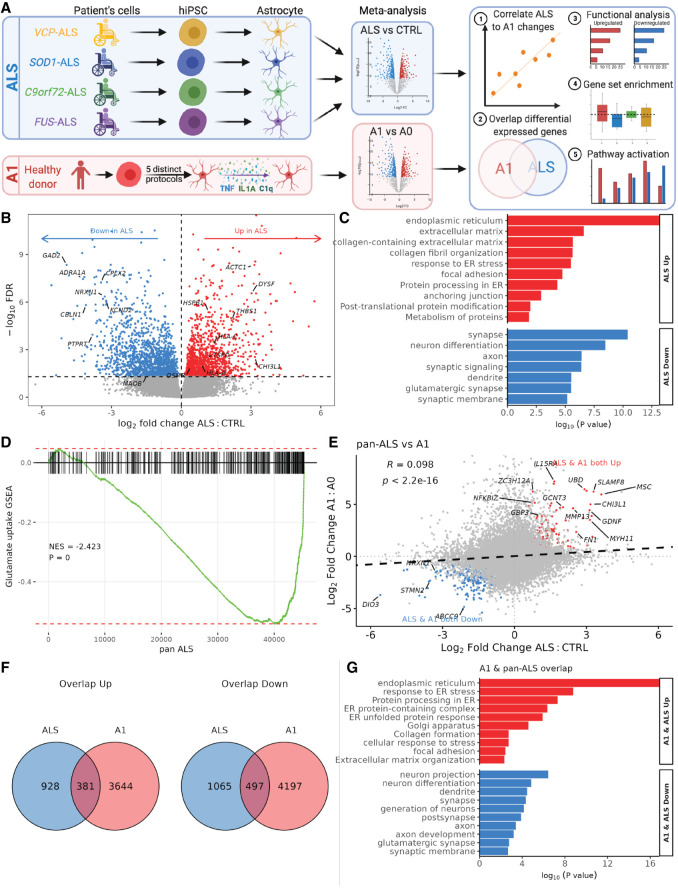
Meta-analysis of ALS hiPSC-astrocytes and correlation with A1 astrocytes. (*A*) Schematic depicting hiPSC-astrocyte differentiation in ALS mutant astrocytes (*VCP*, yellow; *SOD1*, blue; *C9orf72*, green; *FUS*, purple) and A1 hiPSC-astrocytes from five distinct differentiation protocols (red). Astrocytes underwent RNA sequencing and were analyzed for differential expression, with meta-analyses of ALS mutants (blue, *top*) and A1 astrocytes (red, *bottom*). We then ran a downstream bioinformatic analysis by mapping each gene's expression change in ALS versus control with that of A1 versus A0. (*B*) Volcano plot showing log_2_ fold change in differential gene expression from the ALS meta-analysis for pan-ALS versus control astrocytes. Genes with significantly (FDR < 0.05) increased expression are shown in red, and those decreased in expression are shown in blue. (*C*) GO terms enriched in up-regulated (red) and down-regulated (blue) differentially expressed genes in pan-ALS versus control astrocytes. (*D*) Gene set enrichment analyses (GSEA) plot for glutamate uptake in pan-ALS astrocytes. (NES) Normalized enrichment score. Enrichment *P*-value < 1 × 10^10^. (*E*) Scatterplot of log_2_ fold changes in gene expression in pan-ALS versus control astrocytes (*x*-axis) against A1 versus A0 astrocytes (*y*-axis). Black dashed line indicates linear regression correlation (Pearson's correlation R = + 0.1). Overlapping differentially expressed genes are colored red (up-regulated) and blue (down-regulated). (*F*) Venn diagram depicting the overlap of up-regulated (*left*) or down-regulated (*right*) genes (FDR < 0.05) in ALS versus control (blue) and A1 versus A0 (red). (*G*) Bar graph showing curated overrepresented functional categories (FDR < 0.05) by GO analysis of the genes commonly up-regulated (red) and co-down-regulated (blue) between A1 and ALS astrocytes.

To identify pan-ALS transcriptomic perturbations, we performed a meta-analysis of *VCP*, *C9orf72*, *SOD1*, and *FUS* mutant hiPSC-derived astrocytes, accounting for batch effects between data sets (totaling 13 mutant lines and 14 control lines) (Fig. 2A). The meta-analytic approach has the advantage of increasing power and precision to identify gene expression changes that are common across ALS mutations but not detectable in the individual RNA-seq data sets (Rau et al. 2014). We revealed substantial changes in gene expression between pan-ALS and control astrocytes, with 1309 up-regulated and 1562 down-regulated genes (FDR < 0.05) (Fig. 2B). GO analysis of differentially expressed genes revealed that those up-regulated in ALS were enriched in endoplasmic reticulum, extracellular matrix, protein metabolism, and adhesion—processes relevant to reactive transformation. Conversely, genes decreased in expression in ALS were overrepresented by synaptic (e.g., glutamatergic synapse) and neuronal development functions (Fig. 2C). GSEA further confirmed that genes involved in glutamate uptake were significantly decreased in expression in ALS astrocytes (including *GAD2*, *ADRA1A*, *CBLN1*, *PTPRT*, *CACNG3*, *CPLX2*, *NRXN1*, *KCND2*, *SHISA6*, *ELAVL4*, *GRIN1*, *GRIN2B*; NES −2.5, *P* < 1 × 10^−10^) (Fig. 2D). Taken together, these findings indicate that ALS mutant astrocytes increase the expression of genes regulating reactive transformation while concomitantly down-regulating genes involved with neuronal support. These meta-analysis results can be browsed in the interactive web application at https://shiny.crick.ac.uk/ALS_reactive_astrocytes_meta/.

To assess whether ALS astrocytes display shared gene expression changes with A1 astrocytes, we next compared the pan-ALS transcriptomic signature with treated A1-reactive astrocytes. We used five hiPSC-derived astrocyte protocols, each with TNF-, IL1A-, and C1q (A1)-treated and vehicle (A0)-treated samples from Barbar et al. (2020) (one protocol: three A1 and three A0 lines) and Leng et al. (2021) (four protocols: each with three A1 and three A0 lines) (Supplemental Fig. S4A,B). A1 gene expression changes were defined as treated versus untreated samples, accounting for differences between protocols and cell lines. By correlating pan-ALS and A1 differential gene expression changes, we found a significant positive association (Pearson's R = +0.1, *P* < 2.2 × 10^16^) (Fig. 2E), consistent with shared transcriptional alterations between ALS and A1 astrocytes. Of the 1309 genes significantly (FDR < 0.05) up-regulated in ALS astrocytes, 381 (29.1%) were also significantly up-regulated in A1 astrocytes (Fisher's exact test *P* = 4 × 10^141^) (Fig. 2F). Similarly, of the 1562 genes significantly down-regulated in ALS astrocytes, 497 (31.8%) were also down-regulated in A1 astrocytes (*P* = 5.4 × 10^175^) (Supplemental Table S4). GO analysis of these overlapping differentially expressed genes revealed shared enriched processes between ALS and A1 astrocytes, with up-regulation of endoplasmic reticulum, extracellular matrix, and adhesion, whereas genes decreased in expression in ALS and both A1 astrocyte data sets were enriched for synaptic and neuronal development functions (Fig. 2G).

To determine whether the A1 similarities in our ALS hiPSC meta-analysis are triggered by ALS-linked mutations, we next examined a microarray data set of human fibroblasts directly converted to astrocytes (transdifferentiated) from patients with sporadic ALS with no known ALS mutations (two sporadic and two control lines) (Ferraiuolo et al. 2016). Comparing sporadic ALS versus control astrocytes revealed no differentially expressed genes at FDR < 0.05. Using a more lenient threshold of an unadjusted *P*-value < 0.05, we identified 45 up-regulated and 29 down-regulated genes, with no GO terms enriched. GSEA of glutamate uptake revealed a nonsignificant increase in sporadic ALS astrocytes (NES +1.2, enrichment *P* = 0.08) (Supplemental Fig. S5A). Comparing log_2_ fold changes across all genes in sporadic ALS with the mutation-carrying pan ALS (meta-analysis) astrocytes revealed an overall negative correlation (R = −0.1, *P* < 2.2 × 10^16^), with only a single overlapping differentially expressed gene (*NCKAP5*) (Supplemental Fig. S5B). Correlating sporadic ALS astrocytes with A1 astrocyte gene expression changes showed a weak negative correlation (R = −0.02, *P* = 0.053), sharing only three up-regulated and two down-regulated genes (Supplemental Fig. S5C,D). Furthermore, we found only minimal corresponding changes in the reactive markers from Liddelow et al. (2017) between A1 and sporadic astrocytes (Supplemental Fig. S6B). These findings suggest not only that the transcriptomes of sporadic ALS astrocytes differ from familial ALS mutation-linked astrocytes, but also that they do not recapitulate the global gene expression changes observed in A1-treated astrocytes when in monoculture. This raises the possibility that the A1 signature observed in the ALS mutant astrocyte meta-analysis is triggered by ALS-linked mutations themselves.

### ALS astrocytes display a cluster of contrasting gene expression changes compared to protective reactive astrocytes

**Figure 3.**
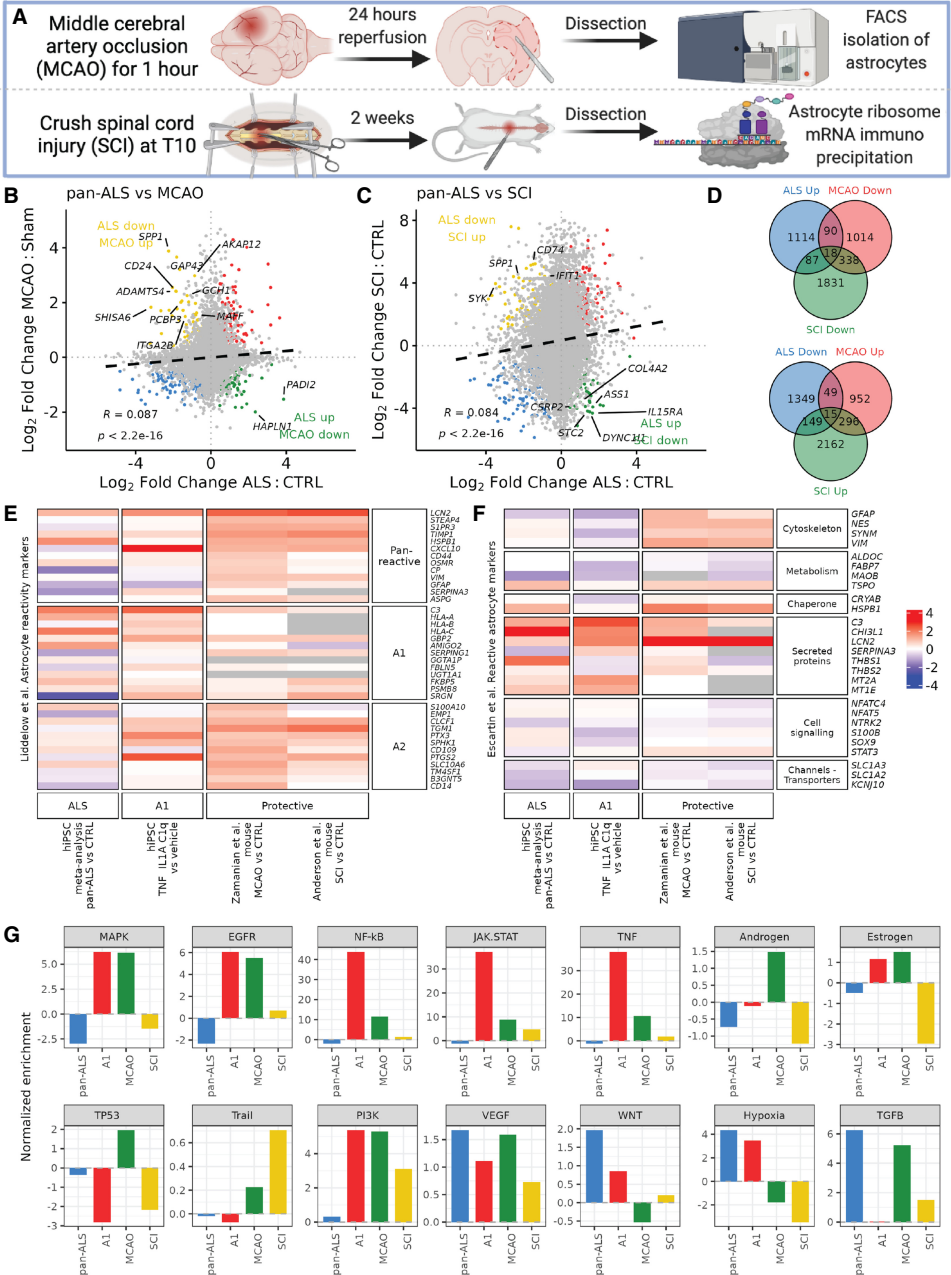
hiPSC ALS astrocytes show convergent and divergent changes compared to protective astrocytes. (*A*) Schematic showing mouse models of protective astrocytes. *Top* row represents a middle cerebral artery occlusion (MCAO) model followed by dissection and astrocyte FACS isolation before RNA sequencing. *Bottom* row shows spinal cord injury (SCI) followed by scar dissection and astrocyte ribosome–associated RNA hemagglutinin immunoprecipitation. (*B*,*C*) Scatterplots of log_2_ fold changes in gene expression in pan-ALS versus control astrocytes (*x*-axis) against MCAO versus sham astrocytes (*B*; *y*-axis) and SCI versus control astrocytes (*C*). Black dashed line indicates linear regression correlation (Pearson's correlation, both R = + 0.09). Overlapping differentially expressed genes are colored red (up-regulated) and blue (down-regulated). Genes increased in ALS but decreased in the protective astrocytes are shown in green, whereas genes decreased in ALS and increased in protective astrocytes are yellow. (*D*) Venn diagram depicting the number of differentially expressed genes (FDR < 0.05) increased in ALS but decreased in the protective astrocytes (*top*) and genes decreased in ALS but increased in protective astrocytes (*bottom*). (*E*,*F*) Heatmaps of Liddelow et al. (2017) pan-reactive, A1- and A2-specific astrocyte reactivity markers (*E*) and (Escartin et al. 2021) markers of reactive astrocytes (*F*; rows) in the hiPSC ALS astrocyte meta-analysis, A1 (TNF, IL1A, C1q) protocols and protective (MCAO and SCI) data sets (columns). Color intensity represents the scaled differential gene expression log_2_ fold changes in pan-ALS versus control; A1 versus A0, MCAO versus sham, and SCI versus control astrocytes. For ALS individual data sets, see Supplemental Figure S3. (*G*) PROGENy signaling pathway activity normalized enrichment scores (*y*-axis) in pan-ALS (blue), hiPSC A1 (red), MCAO (green), and SCI (yellow) astrocytes.

Given that the down-regulated genes in ALS astrocytes are involved with protective astrocyte functions, we next compared ALS astrocyte gene expression changes with that of two mouse models that show protective astrocyte gene expression profiles, characterized by high levels of neurotrophic and axon growth factors (Fig. 3A). These included astrocytes isolated 24 h following MCAO (five MCAO lines and four sham lines) (Zamanian et al. 2012) and scar-forming astrocytes following SCI (seven SCI lines and four control lines) (Anderson et al. 2016). Correlating pan-ALS gene expression changes with both of these data sets revealed positive correlations (MCAO, R = + 0.09; SCI, R = +0.08; both, *P* < 2.2 × 10^16^), suggesting that global gene expression changes tend to converge between ALS and these models, which may represent largely pan-reactive gene expression alterations (Fig. 3B,C). However, of particular interest to us was the substantial number of differentially expressed genes enriched in opposing directions between ALS and both these protective astrocyte models (ALS up & MCAO down: 108 [8.3%], Fisher's exact test *P* = 3.8 × 10^19^; ALS down & MCAO up: 64 [4.9%], *P* = 0.003; ALS up & SCI down: 105 [8.0%], *P* = 4 × 10^13^; ALS down & SCI up: 164 [10.5%], *P* = 1.6 × 10^25^) (Fig. 3D). Eighteen genes were significantly increased in expression in ALS astrocytes but were significantly decreased in both MCAO and SCI astrocytes (including *PYGL*, *VEGFA*, *NR3C2*, *MAGT1*, *RECK*, *CSRP1*; ANOVA test *P* = 6.8 × 10^11^), and 15 genes were decreased in ALS but increased in both protective astrocyte data sets (including *GAP43*, *ADAMTS4*, *SPP1*, *RUBCN*, *GCH1*, *PGD*, *PDE3B*, *DUSP5*, *CD9*; *P* = 1.1 × 10^5^) (Supplemental Table S5). These genes being up-regulated in MCAO and SCI models but down-regulated in ALS astrocytes supports the possibility of a cluster of protective genes that is lost in ALS (and vice versa).

Of the previously reported astrocyte reactivity gene panel of “pan-reactive,” “A1,” and “A2” reactive categories from Liddelow et al. (2017), we observed a positive correlation between ALS and A1 astrocytes (R = +0.13), with convergent expression changes in both ALS and A1 astrocytes of 54% (22/41) of genes (Fig. 3E; Supplemental Fig. S6A). Conversely, we identified only a very weak positive correlation between ALS and MCAO astrocytes (R = +0.01) and between ALS and SCI astrocytes (R = +0.03) (Supplemental Fig. S6C–E). Of note, we found that the A1 astrocyte expression changes themselves were not restricted to “A1” and “pan-reactive” categories, supporting a growing consensus that reactive states exist on a spectrum of heterogeneous context-specific phenotypes, rather than an A1-versus-A2 binary classification (Escartin et al. 2021). Indeed, examining the potential markers of reactive astrocytes from Escartin et al. (2021) revealed increased expression of secreted proteins (except *SERPINA3*) and down-regulation of channel transporters across ALS, A1, and protective astrocytes (Fig. 3F; Supplemental Fig. S6F). Cytoskeleton markers (e.g., *GFAP*) were decreased in the ALS and A1 data sets but increased in the MCAO and SCI models, suggesting that they may play a role in neuroprotection.

To understand whether signaling pathways are activated in ALS and how this relates to A1 and protective astrocytes, we next performed a pathway responsive genes (PROGENy) analysis (Schubert et al. 2018). In ALS astrocytes, the most substantial increase was noted in the profibrotic TGFB signaling pathway, followed by hypoxia, Wnt, and VEGF pathways (Fig. 3G). The most responsive genes within the TGFB pathway included *LTBP2*, *COL1A1*, and *LMCD1* in ALS astrocytes (Supplemental Fig. S7). Comparing ALS to A1 astrocytes revealed common up-regulation of the hypoxia, Wnt, VEGF, and PI3K pathways and co-down-regulation of the TP53 DNA damage and androgen pathways. Comparing both protective astrocyte data sets to the ALS and A1 changes revealed that although the TGFB, VEGF, and PI3K pathways were also up-regulated, we found that hypoxia signaling was decreased, indicating that up-regulation of hypoxia genes might be unique to deleterious reactive astrocytes. Although the NF-kB, JAK-STAT, and TNF pathways were decreased in ALS, they were up-regulated in both A1 and protective astrocytes. Taken together, although many pathways show divergent changes between the ALS and A1 astrocytes, the common up-regulation in hypoxia and Wnt pathways may be in part responsible for ALS astrocytes adopting A1-reactive deleterious alterations.

### Meta-analysis of in vivo mouse models of ALS astrocytes shows resemblance with proinflammatory reactive astrocytes

**Figure 4.**
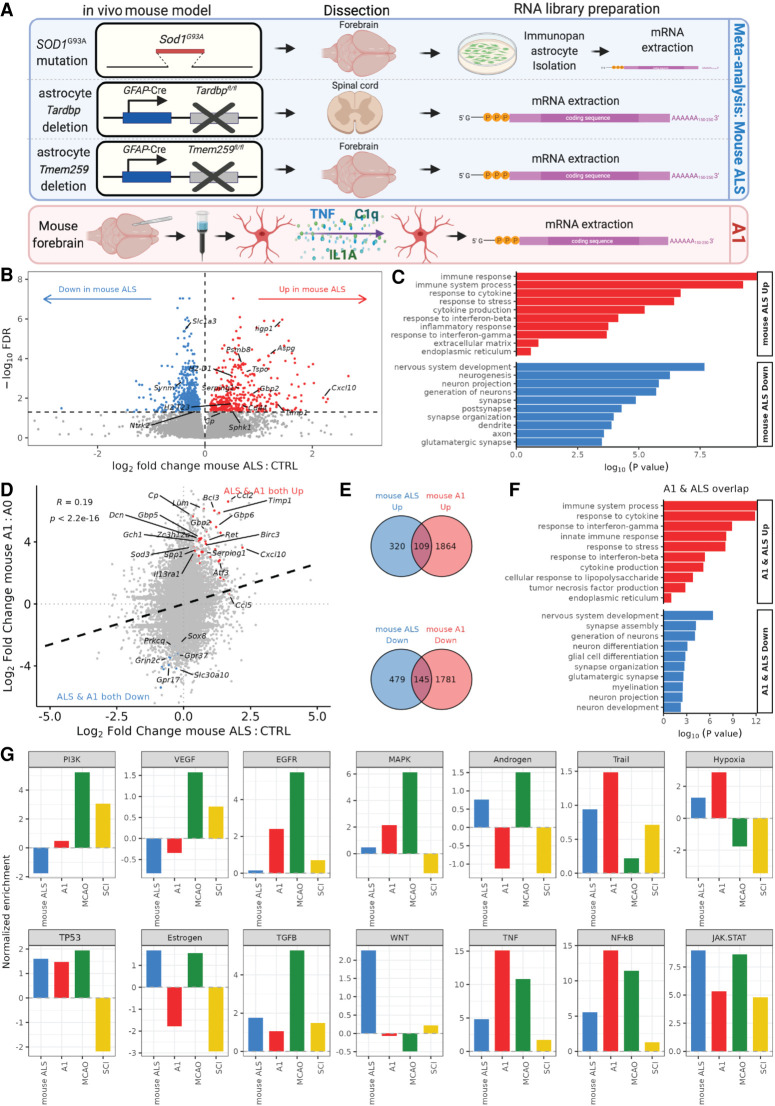
Meta-analysis of astrocyte-specific ALS mouse models and A1 astrocyte correlation. (*A*) Schematic depicting mouse in vivo models: ALS *SOD1^G37R^* mutant mouse forebrain HepaCAM immunopanned isolated and cultured astrocytes in serum-free conditions; astrocyte-specific *Tardbp* deletion using conditional GFAP-Cre recombinase promoter followed by spinal cord dissection; astrocyte-specific *Tmem259* deletion (GFAP-Cre promoter) forebrain dissection; and mouse forebrain isolated astrocytes treated with A1 factors before mRNA library preparation and sequencing. (*B*) Volcano plot showing log_2_ fold change in differential gene expression from the mouse ALS model meta-analysis for mouse ALS versus control. Genes with significantly (FDR < 0.05) increased expression are shown in red, and those decreased in expression are shown in blue. (*C*) GO terms enriched in up-regulated (red) and down-regulated (blue) differentially expressed genes in mouse ALS versus control astrocytes. (*D*) Scatterplot of log_2_ fold changes in gene expression in mouse ALS versus control astrocytes (*x*-axis) against mouse A1 versus A0 astrocytes (*y*-axis). Black dashed line indicates linear regression correlation (Pearson's correlation R = + 0.19). Overlapping differentially expressed genes are colored red (up-regulated) and blue (down-regulated). (*E*) Venn diagram depicting the overlap of up-regulated (*top*) or down-regulated (*bottom*) genes (FDR < 0.05) in mouse ALS versus control (blue) and mouse A1 versus A0 astrocytes (red). (*F*) Bar graph showing curated overrepresented functional categories (FDR < 0.05) by GO analysis of the 109 genes commonly up-regulated (red) and 145 co-down-regulated (blue) between mouse A1 and mouse ALS astrocytes. (*G*) PROGENy signaling pathway activity normalized enrichment scores (NESs) for the mouse ALS (blue), mouse A1 (red), MCAO (green), and SCI (yellow) data sets.

To further explore the molecular mechanisms associated with reactive transformation of ALS astrocytes, we next investigated whether mouse models addressing ALS pathogenesis recapitulate the transcriptomic perturbations seen in ALS and reactive hiPSC-derived astrocytes. We reanalyzed mouse ALS astrocytes carrying the *SOD1^G93A^* transgene mutation (three mutant and three wild-type mice) (Guttenplan et al. 2020), astrocyte-specific knockout of *Tardbp* (using GFAP-Cre recombinase promoter *Tardbp* knockout; four knockout and four control mice) (Peng et al. 2020), and motor cortex astrocyte-specific *Tmem259* knockout (five knockout and five control mice) (Jiang et al. 2019) (Fig. 4A). In all three mouse models, A1 markers have been experimentally shown to be up-regulated, and A1-like phenotypes have been reported. We first corroborated that gene expression changes in these models resemble the global transcriptome of reactive astrocytes, with up-regulation of genes involved in the stress response and extracellular matrix and down-regulation of neuronal- and synapse-related genes (Supplemental Fig. S8A–F). To investigate whether these mouse ALS astrocyte-centered models show differential expression within the same genes, we overlapped genes significantly changed in expression between *Sod1*, *Tardbp*, and *Tmem259* models (Supplemental Fig. S8G; Supplemental Table S6). Insulin-like growth factor binding protein 4 (*Igfbp4*) was significantly increased in expression in all three ALS mouse astrocyte models, whereas *Tmem132b* was significantly decreased in expression.

To identify transcriptomic perturbations across the mouse ALS models, we performed a meta-analysis of *SOD1^G93A^*, *Tardbp*, and *Tmem259* knockout mice, adjusting for batch effects between data sets (total 12 ALS and 12 control mice). This identified 429 up-regulated and 624 down-regulated genes in the mouse ALS models (FDR < 0.05) (Fig. 4B). GO terms enriched among up-regulated genes in ALS included immune response and inflammation, whereas down-regulated genes were overrepresented by neuron development and synapse terms (Fig. 4C). GSEA revealed a nonsignificant overall decrease in the expression of the glutamate uptake gene set in models of mouse ALS astrocytes (NES −0.8, *P* = 0.96), although we did note significant decreases in many individual glutamate transporter-related genes, including *Slc1a3*, *Il1rap*, *Eps15*, *Tnr*, *Grin2c*, *Sptbn2*, *Septin11*, *Sptbn1*, and *Arhgap39* (Supplemental Fig. S8H). This indicates that these mouse models of ALS astrocyte pathogenesis show a generalized transcriptomic signature of heightened inflammation with concomitant attenuated astrocyte supportive functions, consistent with the hiPSC ALS meta-analysis.

To examine whether there was conservation in the transcriptomic signature between human and mouse, we compared the hiPSC ALS and mouse ALS astrocyte meta-analyses. Correlating the hiPSC ALS with mouse ALS model gene expression changes revealed a weak positive association (Pearson's R = +0.05, *P* = 1.6 × 10^10^) (Supplemental Fig. S9A). Of the 429 genes significantly (FDR < 0.05) up-regulated in mouse ALS models, 41 (9.6%) were also significantly up-regulated in hiPSC ALS astrocytes (Fisher's exact test *P* = 3.2 × 10^15^) (Supplemental Fig. S9B). Similarly, of the 624 genes significantly down-regulated in mouse ALS models astrocytes, 83 (13.3%) were also down-regulated in hiPSC ALS astrocytes (*P* = 2.1 × 10^34^) (Supplemental Table S7). GO analysis of these overlapping differentially expressed genes identified shared enriched processes between human and mouse ALS astrocyte meta-analyses, with co-up-regulation of extracellular matrix and immune response pathways and co-down-regulation of neuronal and synapse genes (Supplemental Fig. S9C). These findings suggest that both the human and mouse ALS astrocyte models are characterized by a gain of inflammation and loss of supportive functions.

To further investigate whether mouse ALS models show shared transcriptome perturbations with A1 astrocytes, we compared the mouse ALS transcriptomic signature with treated A1-reactive mouse astrocytes from Guttenplan et al. (2020) (three TNF-, IL1A-, and C1q-treated and three vehicle lines). Correlating mouse ALS models and A1 astrocyte gene expression changes revealed a positive association (Pearson's R = +0.19, *P* < 2.2 × 10^16^) (Fig. 4D). Of the 429 genes significantly (FDR < 0.05) up-regulated in mouse ALS astrocytes, 109 (25.4%) were also significantly up-regulated in mouse A1 astrocytes (Fisher's exact test *P* = 2.4 × 10^60^) (Fig. 4E). Likewise, of the 624 genes significantly down-regulated in mouse ALS astrocytes, 145 (23.2%) were also down-regulated in mouse A1 astrocytes (*P* = 7.3 × 10^76^) (Supplemental Table S8). GO analysis of the 109 commonly up-regulated genes was enriched for immune response and cytokine signaling processes, whereas the 145 co-down-regulated genes were overrepresented by neuronal supportive and synaptic functions (Fig. 4F). Correlating mouse ALS models’ astrocyte gene expression changes with the protective MCAO and SCI astrocytes also revealed a positive correlation with MCAO (R = +0.1) (Supplemental Fig. S10A) but a negative correlation with SCI (R = −0.04) (Supplemental Fig. S10B). In line with this, we found that differentially expressed genes enriched in opposing directions overlapped between mouse ALS models and SCI (ALS up & SCI down: 42 [9.7%], *P* = 2.4 × 10^7^; ALS down & SCI up: 166 [26.6%], *P* = 2.6 × 10^76^) but only a modest contrasting overlap between mouse ALS models and MCAO (ALS up & MCAO down: 33 [7.7%], Fisher's exact test *P* = 5.2 × 10^4^; ALS down & MCAO up: 22 [3.5%], *P* = 0.6) (Supplemental Fig. S10C,D). Eight genes were significantly increased in expression in mouse ALS models but significantly decreased in both MCAO and SCI protective astrocytes (*Atp6ap2*, *Ndufv3*, *Prkd1*, *Psenen*, *Slc25a20*, *Suclg1*, *Tlr3*, *Tspan3*; ANOVA test *P* = 0.0005), and 14 genes were decreased in mouse ALS models but increased in both protective astrocyte data sets (including *Adamts4*, *Ddx54*, *Dst*, *Myo5a*, *Nfasc*, *Pdgfa*, *Phldb1*, *Pus7*, *Synm*; *P* = 0.12) (Supplemental Table S9). These results confirm that these mouse ALS astrocyte models recapitulate gene expression changes observed in A1-reactive astrocytes but show contrasting changes in a cluster of genes involved with protective responses.

Signaling pathway analysis revealed that the mouse ALS models led to up-regulation of inflammatory pathways, with the largest increases observed in JAK-STAT, NF-kB, and TNF (Fig. 4G). Mouse ALS and mouse A1 astrocytes shared common up-regulation of these three pathways as well as six others (TGFB, TP53, hypoxia, Trail, MAPK, and EGFR). As with hiPSC-astrocytes, hypoxia signaling was increased in the ALS and A1 astrocytes but decreased in the protective astrocytes. Examining A1, A2, and pan-reactive astrocyte markers revealed a positive association between the mouse ALS models and A1 astrocytes (R = +0.12), with increased expression in both the mouse ALS models and A1 astrocytes in 85% (29/34) (Supplemental Fig. S11A,C). Examining changes in markers of reactive astrocytes from Escartin et al. (2021) revealed similar patterns to human ALS and A1 astrocytes, with increased expression of secreted proteins and down-regulation of channel transporters (Supplemental Fig. S11D). Taken together, this raises the possibility that these mouse ALS models predispose to A1-like reactive transformation, as observed in hiPSC ALS astrocytes.

To establish whether the A1-reactive gene expression changes observed in ALS astrocytes influence mRNA translation, we reanalyzed translating ribosome-bound mRNA sequencing from *SOD1^G37R^* mutant mouse spinal cord astrocytes (TRAP-seq; four *SOD1^G37R^* and six control samples) (Supplemental Fig. S12A; Sun et al. 2015). Comparing translation changes in the *SOD1^G37R^* astrocytes with gene expression changes in A1 hiPSC and mouse as well as protective astrocytes revealed positive associations (hiPSC A1 R = +0.06, mouse A1 R = +0.07, MCAO R = +0.13, SCI R = +0.06) (Supplemental Fig. S12B–E). Overlapping genes significantly increased in translation in *SOD1*^*G37R*^ astrocytes with those increased in both hiPSC and mouse A1-reactive astrocytes, revealing 65/590 (11.0%, ANOVA test *P* = 4.7 × 10^11^) overlapping genes (Supplemental Fig. S12F). Of the genes showing decreased translation in *SOD1^G37R^*, 40/452 (8.8%, *P* = 5.9 × 10^9^) displayed decreased expression in both A1 data sets (Supplemental Table S10). GO analysis of these overlapping *SOD1^G37R^* translated and A1 expressed genes revealed up-regulation of immune response processes, including cytokine response, TNF, and interferon signaling (Supplemental Fig. S12G). Of the A1, A2, and pan-reactive panels, we observed increased expression in both *SOD1^G37R^*- and A1-treated astrocytes in 93.8% (30/32) (Supplemental Fig. S11B–D). Thus, the results from Sun et al. (2015) further corroborate not only the existence of A1-like gene expression signatures in ALS astrocytes but that the transcriptomic changes correspond with alterations at the translatomic level.

## Discussion

**Figure 5.**
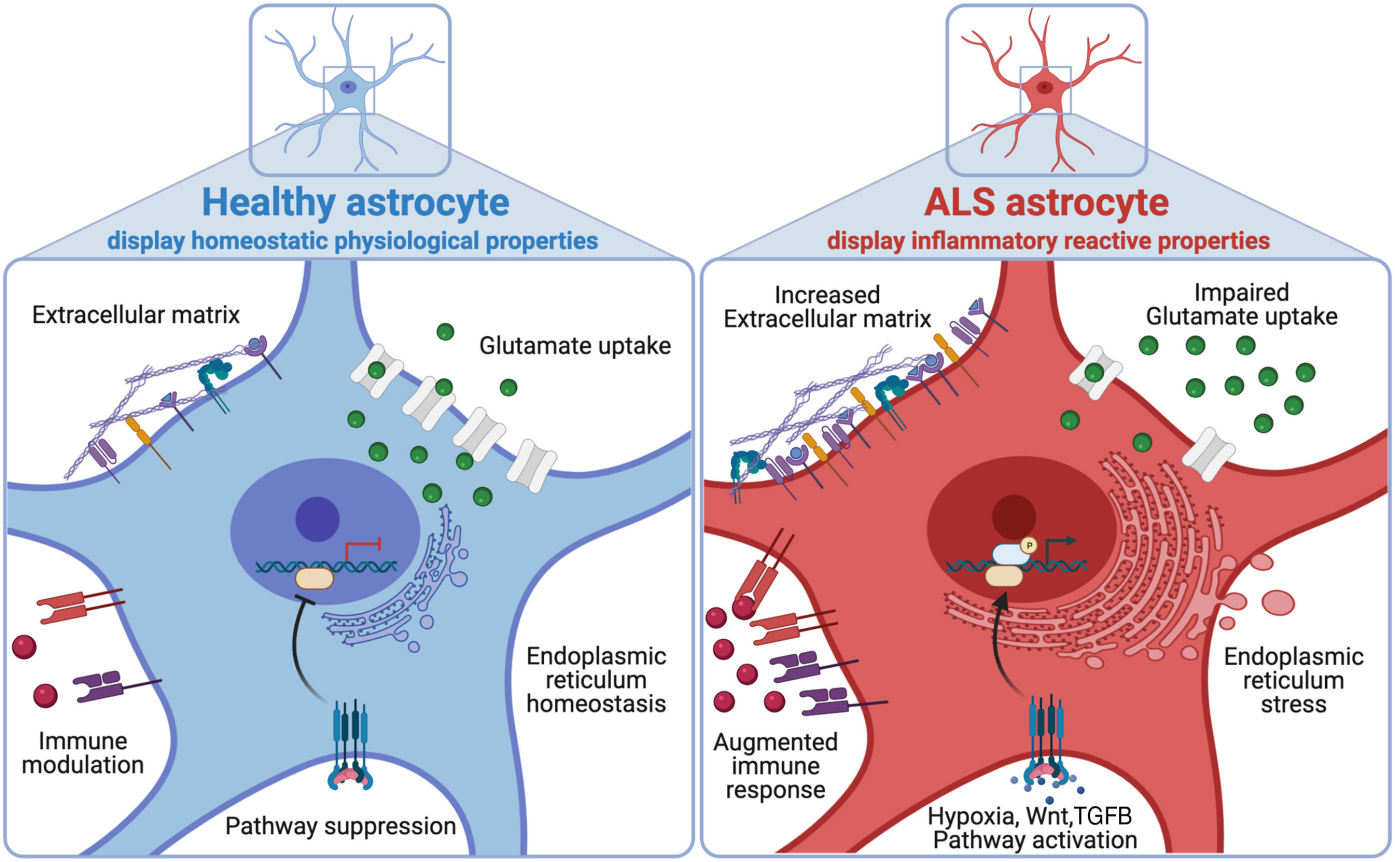
Working model. Multi-omic signatures of healthy astrocytes (*left*, blue) and ALS astrocytes (*right*, red).

Astrocytes have been implicated in ALS pathogenesis, but whether ALS astrocytes show similarities with the neurotoxic A1-reactive inflammatory state is unclear (Nagai et al. 2007; Yamanaka et al. 2008; Meyer et al. 2014; Liddelow et al. 2017). Here, we used transcriptomics, proteomics, and a glutamate uptake assay of *VCP*-mutant hiPSC-derived astrocytes combined with meta-analysis of all publicly available ALS astrocyte sequencing data to systematically identify genes that are perturbed across ALS astrocytes. By comparing and contrasting ALS gene expression changes to distinct models of inflammatory reactive astrocytes, including the A1 (TNF, IL1A, and C1q treated) and protective (MCAO, SCI) data sets, we reveal widespread changes that correspond with inflammatory reactive gene expression and signaling pathway activity (Fig. 5). To provide access to both the human and mouse transcriptome data, we have established an interactive web application (https://shiny.crick.ac.uk/ALS_reactive_astrocytes_meta/).

We identified overlapping signatures between inflammatory reactive astrocytes and ALS mutant astrocytes, characterized by common up-regulation of immune-related genes and concomitant down-regulation of synaptic and neuronal genes. However, it should be noted that we generally found only weak positive correlations between ALS and inflammatory reactive gene expression changes (R ∼ +0.1). Given the increasingly recognized heterogeneity in astrocyte-reactive states, this is unsurprising and supports a recent consensus statement highlighting the shortcomings of binary classifications of reactive astrocytes into A1 (detrimental) versus A2 (protective), which oversimplify the diverse multidimensional inflammatory reactive states (Escartin et al. 2021). Furthermore, we identified up-regulation of some A1 markers among protective astrocyte data sets (MCAO, SCI) as well as up-regulation of some A2 markers in the A1 data sets (Fig. 3E), highlighting the importance of examining multiple molecular markers of reactivity with varied functions (Fig. 3F) rather than just the A1 and A2 marker genes.

Extracellular glutamate homeostasis is of crucial importance in the CNS, where astrocytic glutamate uptake helps maintain tight glutamate regulation, avoiding excitotoxicity (Rothstein et al. 1996). In *VCP*-mutant astrocytes, we identified decreased functional ability to perform glutamate uptake and a decrease in proteins involved in glutamate uptake by mass spectroscopy. In both human and mouse ALS astrocyte meta-analyses, we found that down-regulated genes were overrepresented by “glutamatergic synapse,” and GSEA further revealed decreased expression across the glutamate uptake gene set. After filtering ALS down-regulated genes to those also decreased in A1 astrocytes, we found that “glutamatergic synapse” remained enriched in both human and mouse analyses. This is consistent with other studies of the A1-reactive state, in which glutamate receptors and transporters have been shown to be down-regulated (Barbar et al. 2020). Furthermore, astrocyte-specific *Tmem259* deletion induced extracellular glutamate accumulation and excitotoxicity with increased expression of A1 reactivity markers (Jiang et al. 2019). Although we found that *C9orf72* mutant astrocytes had decreased expression of the glutamate uptake gene set (Supplemental Fig. S3G), they were previously reported to have no change in their functional rate of glutamate uptake (Birger et al. 2019); however, this may be owing to technical reasons as astrocytes were assayed at 60 min rather than at 3 h, as used in this study and the one by Barbar et al. (2020). Together, these findings raise the question of whether A1-like changes in ALS are a reactive response to impaired glutamate uptake and subsequent toxicity or whether A1 reactivity itself results in impaired glutamate uptake—possibilities that are non-mutually-exclusive.

Contrary to ALS mutation-carrying astrocytes, we found that microarray of sporadic ALS transdifferentiated astrocytes with no known mutations did not correlate with global A1 changes and showed a nonsignificant increase in the expression of glutamate uptake components (Ferraiuolo et al. 2016). It is noteworthy, however, that the comparison between the sporadic ALS data set and our meta-analyses is restricted by a number of limitations, including differences between (1) microarray and RNA-seq, (2) the protocols used to generate astrocytes (transdifferentiation vs. hiPSC), and (3) the presence of fetal bovine serum in the media to generate sporadic ALS astrocytes, which affects astrocyte reactivity (Foo et al. 2011). Nevertheless, these findings need to be reconciled with studies reporting that sporadic ALS astrocytes participate in neuronal degeneration (Haidet-Phillips et al. 2011; Meyer et al. 2014; Re et al. 2014; Qian et al. 2017), which is an important avenue of future research. It remains unclear whether sporadic ALS hiPSC-derived enriched astrocytes can exert non-cell-autonomous toxicity to motor neurons through either conditioned medium or physical coculture.

Following up on the immune-related enriched terms from the ALS meta-analysis, we identified activation of reactive signaling in the hypoxia, Wnt, VEGF, and PI3K pathways and co-down-regulation of the TP53 DNA damage pathway in ALS and A1 hiPSC-astrocytes. We found that hypoxia signaling was increased in both A1 and ALS astrocytes but decreased in the protective astrocyte models, suggesting that hypoxia signaling may be unique to deleterious reactive astrocytes. Hypoxia has previously been implicated in neurodegenerative diseases, including in ALS, where it has been suggested to aggravate motor neuron degeneration (Oosthuyse et al. 2001; Sato et al. 2012; Kim et al. 2013; Nagara et al. 2013). Although the hypoxic response is initially neuroprotective, prolonged hypoxia signaling can trigger neuroinflammation and oxidative stress, leading to neuronal dysfunction (Allen et al. 2020). Astrocytes respond to hypoxia by increasing blood–brain barrier permeability, neuroinflammation, and cytokine secretion (Marina et al. 2016; Samy et al. 2018). Thus, hypoxia signaling may provide an attractive therapeutic target in ALS to prevent deleterious reactive astrocyte states.

There was partial conservation in the gene expression changes between human and mouse models of ALS astrocytes, with similar GO term enrichment. However, the correlation was particularly weak (R = +0.05), suggestive of interspecies and experimental context differences between the hiPSC and mouse astrocyte data sets (Oberheim et al. 2012; Zhang et al. 2016). Indeed, although hiPSC-derived astrocytes were critical to our meta-analysis, their relative fetal maturation status limits the study of age-related effects on astrocyte reactivity (Clarke et al. 2018). The hiPSC-astrocyte monoculture is also devoid of other cell types that interact in vivo, such as degenerating neurons or microglia that contribute to astrocyte-reactive transformation. Additionally, the mouse models show important differences (e.g., in vitro culture vs. in vivo isolation; forebrain vs. spinal cord) (depicted in Figs. 3A, 4A), which may impact on astrocyte reactivity.

Overall, our study provides new insights into characterizing reactive transformation of ALS astrocytes. Astrocyte functions help regulate a healthy CNS environment, enabling optimal neuronal function; however, ALS astrocytes undergo reactive changes, which may be an initial protective mechanism but then become toxic with time with loss of homeostatic functions. The key reactive astrocyte regulators that drive ALS pathogenesis remain to be elucidated, as are perturbed astrocyte functions that are critical to restore in ALS. Further mechanistic dissection of these pathways will pave the way for an improved understanding and for therapeutic targets in ALS, as well as other neurological diseases, that manifest inflammatory astrocyte reactivity.

## Methods

### hiPSC-astrocyte culture

All cell cultures were maintained at 37°C and 5% carbon dioxide. hiPSCs were maintained on Geltrex (Thermo Fisher Scientific) with Essential 8 medium media (Thermo Fisher Scientific) and passaged using EDTA. Generation of hiPSC-astrocytes was performed using a previously described protocol (Hall et al. 2017). Briefly, after neural conversion (7 d in a chemically defined medium containing 1 μM dorsomorphin [Millipore], 2 μM SB431542 [Tocris Bioscience], and 3.3 μM CHIR99021 [Miltenyi Biotec]), neural precursors were patterned for 7 d with 0.5 μM retinoic acid and 1 μM purmorphamine, followed by a 4-d treatment with 0.1 μM purmorphamine. After a propagation phase (>60 d) with 10 ng/mL FGF-2 (Gibco), they were terminally differentiated to astrocytes in the presence of BMP4 (10 ng/mL, R&D) and LIF (10 ng/mL, Sigma-Aldrich) for 23 d followed by 7 d in N2B27.

### Glutamate uptake assay

hiPSC-derived astrocytes were incubated in Hank's balanced salt solution (HBSS) buffer without calcium and magnesium for 30 min before incubation for 3 h in HBSS with calcium and magnesium and 100 μM glutamate. Samples were measured by a colorimetric glutamate assay kit (Abcam Ab83389) according to the manufacturer's instructions. Samples without glutamate were run as negative control, as well as 1 mM PDC-treated cells and cells cultured in Na^+^-free media. Samples were measured using a spectrophotometer at 450 nm. For *VCP*-mutant and control comparison, values were normalized to confluency by a nuclear stain, and data were plotted in R (R Core Team 2017). To normalize for variation between each of one to four experimental repeats, fold changes were calculated by dividing the mean of three technical replicates over the control samples in each experimental repeat. The Wilcoxon test was used to assess statistical significance.

### Immunolabeling and image analysis

Samples were fixed in 4% paraformaldehyde and then blocked in 5% bovine serum albumin (BSA) in PBS, 0.3% Triton X-100 for 1 h at room temperature. Primary antibody chicken anti-GFAP (Abcam ab4674) 1:500 incubation was performed overnight at 4°C in 5% BSA and 0.3% Triton X-100 in PBS, followed by incubation with secondary goat anti-chicken Alexa fluor fluorescent antibody (Thermo Fisher Scientific A11039) in 5% BSA and 0.3% Triton X-100 in PBS for 1 h at room temperature. 4′,6-Diamidino-2-phenylindole (DAPI) was used as a nuclear counterstain (100 ng/mL). Images were acquired using the Opera Phenix high-content screening system (PerkinElmer) with the 40× water objective as confocal z-stacks with a z-step of 1 μm and were processed as maximum projections. For each well, 10 fields were acquired and analyzed using the Columbus image analysis system (PerkinElmer). Morphological analysis was performed on GFAP^+^ astrocytes. The cell outline was used to measure cell roundness, length, width, and area. DAPI stain was used to measure nuclear roundness, length, width, and area.

### Proteomics

Astrocytes were fractionated into nuclear and cytoplasmic fractions, and protein samples were then reduced, alkylated, and acetone-precipitated overnight. Each protein pellet was resuspended in 1 M guanidine hydrochloride and 100 mM HEPES. Proteins were tryptically digested overnight at 37°C with mixing. Digested samples were acidified and then stored at −80°C. Each sample was split into triplicates and loaded onto prepared Evotips. Samples were analyzed using an Evosep 15-cm column and an Orbitrap Fusion mass spectrometer operating in data-dependent acquisition mode. A 44-min universal (OT/IT) method was used. Raw files were analyzed with MaxQuant v1.6.12.0 using the LFQ algorithm against a 2020 Swiss-Prot *Homo sapiens* protein database.

### Statistical analyses

Publicly available RNA sequencing data sets of hiPSC-astrocytes were identified using a search strategy including keywords relating to ALS, astrocytes, or reactive transformation. We systematically reviewed RNA sequencing databases, including NCBI Gene Expression Omnibus (GEO; https://www.ncbi.nlm.nih.gov/geo/), NCBI Sequence Read Archive (SRA; https://www.ncbi.nlm.nih.gov/sra), EBI ArrayExpress (https://www.ebi.ac.uk/arrayexpress/), European Nucleotide Archive (ENA; https://www.ebi.ac.uk/ena/browser/home), and Synapse (https://www.synapse.org) and manually searched reference lists of relevant studies up to October 2021. The 16 publicly available sequencing data sets used in this are listed in Table 1. Mass spectrometry data are deposited at the ProteomeXchange Consortium (http://proteomecentral.proteomexchange.org) via the PRIDE partner repository with the data set identifier PXD022604. An interactive web resource for browsing the processed sequencing data is available for exploration at https://shiny.crick.ac.uk/ALS_reactive_astrocytes_meta/.

For all RNA sequencing data sets, reads were processed using the nfcore/rna-seq v3.0 pipeline (Ewels et al. 2020). Raw reads underwent adaptor trimming with Trim Galore! (https://www.bioinformatics.babraham.ac.uk/projects/trim_galore/), removal of ribosomal RNA with SortMeRNA (Kopylova et al. 2012), alignment to Ensembl GRCh38.99 human reference genome using the splice-aware aligner STAR v2.7.1 (Dobin et al. 2013), and BAM-level quantification with Salmon (Patro et al. 2017). Reads from mouse models were aligned to the Ensembl GRCm38.101 reference genome. Detailed quality control of aligned reads was assessed using the FastQC (https://www.bioinformatics.babraham.ac.uk/projects/fastqc/), RSeQC (Wang et al. 2012), Qualimap (Okonechnikov et al. 2016), dupRadar (Sayols et al. 2016), Preseq (Daley and Smith 2013), and MultiQC (Ewels et al. 2016) tools. Differential gene expression was measured using DESeq2 (Love et al. 2014) at the gene-level in R v4.0.3 (R Core Team 2017).

We confirmed the astrocyte identities of hiPSC-astrocytes by clustering with RNA sequencing studies of cell subpopulations isolated and purified from the human brain from Zhang et al. (2016). Furthermore, by clustering with regionally specified hiPSC-derived astrocytes, we confirmed the ventral spinal cord identities of our hiPSC-astrocytes (Bradley et al. 2019). Meta-analysis results of ALS hiPSC-astrocytes (*VCP*, *SOD1*, *FUS*, and *C9orf72*) were generated by comparing the ALS mutant versus control groups, controlling for data set variation in the design formula (∼data set + group), thereby increasing the sensitivity for identifying differences owing to ALS. Similarly, meta-analysis of mouse models of ALS astrocytes (*SOD1^G93A^*, *Tardbp*, and *Tmem259* depletion) was calculated by comparing the knockout (or mutant) versus control groups, controlling for data set variation in the design formula (∼data set + group). Results for proinflammatory factor–treated A1 astrocytes were created by comparing A1 (TNF-, IL1A-, and C1q-treated) versus A0 (untreated) samples, among five distinct protocols from Barbar et al. (2020) and Leng et al. (2021). The samples from the protocol named Fernandopulle were removed because this was an outlier protocol on the PCA and A1 samples clustered together with vehicle-treated samples. Effects owing to the protocol as well as the cell line (each cell line had a treated and untreated sample) were controlled for using the design formula. The protective astrocyte studies were contrasted as MCAO versus sham (24-h samples) and SCI versus control. A gene was considered significantly differentially expressed if the false-discovery rate (FDR) was < 0.05. To identify enriched functional pathways, GO enrichment was performed using *g:Profiler2*. Significantly overrepresented (FDR < 0.05) up- and down-regulated differentially expressed genes were used as input, with all measured genes used as background. In the bar charts, the top significant GO terms were manually curated by removing redundant terms. GSEA was performed using the FGSEA package (Korotkevich et al. 2021) on the gene sets for glutamatergic synapse components. Overlap between two lists of genes was tested using the Fisher's exact test, whereas overlap between three lists was tested using the listOverlaps function that uses the ANOVA test. PROGENy was used to estimate pathway deregulation (Schubert et al. 2018). PROGENy scores were calculated using the PROGENy weights from the differential expression results, and scores were scaled to their respective null distribution to obtain a normalized pathway score.

Mass spectrometry proteomics data were analyzed using DEP (v1.11.0) (Zhang et al. 2018) on MaxQuant results. Data were filtered, normalized, and imputed using default parameters. Differential enrichment analysis was performed, comparing nuclear and cytoplasmic *VCP*-mutant fractions with control fractions using protein-wise linear models combined with Bayes statistics that uses *limma*. A protein was considered significantly differentially expressed when *P* < 0.05. Schematics were created with BioRender. All error bars in the boxplots shown represent 1.5 times the interquartile range.

### Compliance with ethical standards

Informed consent was obtained from all patients and healthy controls contributing to hiPSC lines used in this study. Experimental protocols were all performed according to approved regulations and guidelines by UCLH's National Hospital for Neurology and Neurosurgery and UCL Queen Square Institute of Neurology joint research ethics committee (09/0272).

## Data access

All raw and processed sequencing data generated in this study have been submitted to the NCBI Gene Expression Omnibus (GEO; https://www.ncbi.nlm.nih.gov/geo/) under accession number GSE160133. Code is available at GitHub (https://github.com/ojziff/ALS_reactive_astrocytes_meta) and as Supplemental Code.

## Supplementary Material

Supplemental Material
