# Supplementary material for: Meta-analysis of human and mouse ALS astrocytes reveals multi-omic signatures of inflammatory reactive states
Source: Genome Res. 2022 Jan;32(1):71–84. doi: 10.1101/gr.275939.121 (PMC8744676; doi:10.1101/gr.275939.121)
Supplement: Supplemental Material [file supp_32_1_71__DC1.html]

Meta-analysis of human and mouse ALS astrocytes reveals multi-omic signatures of inflammatory reactive states — Supplemental Material 

# Meta-analysis of human and mouse ALS astrocytes reveals multi-omic signatures of inflammatory reactive states

## Supplemental Material

- Supplemental\_Code.zip
- Supplemental\_Figures.pdf
- Supplemental\_Tables.xlsx
