# Supplementary figures and images for: Meta-analysis of human and mouse ALS astrocytes reveals multi-omic signatures of inflammatory reactive states
Source: Genome Res. 2022 Jan;32(1):71–84. doi: 10.1101/gr.275939.121 (PMC8744676; doi:10.1101/gr.275939.121)

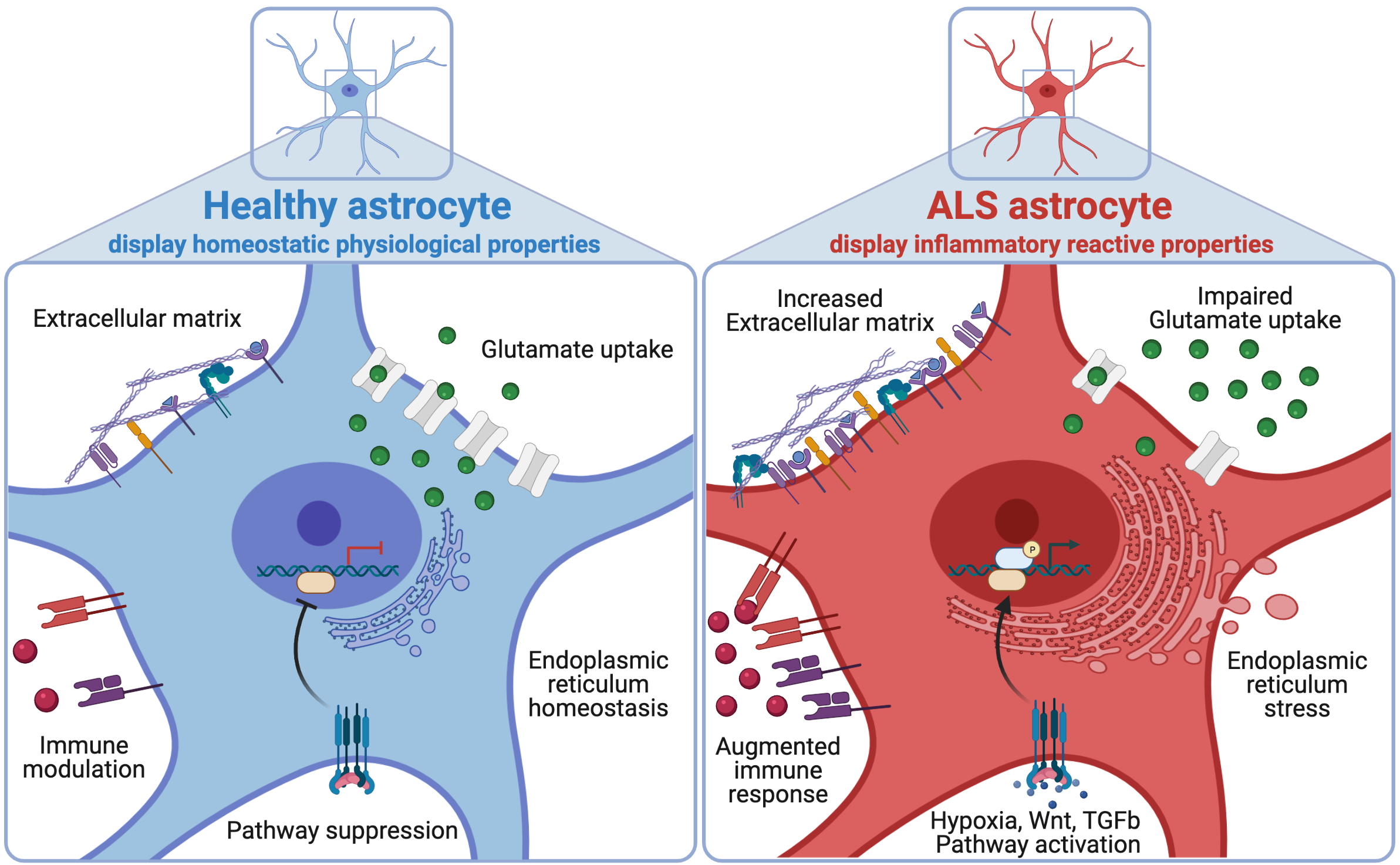

Supplement: Supplemental Material [file supp_gr.275939.121_Supplemental_Code.zip › ALS_reactive_astrocytes_meta-main/working_model.fig5.png]
