# Supplementary material for: Meta-analysis of human and mouse ALS astrocytes reveals multi-omic signatures of inflammatory reactive states
Source: Genome Res. 2022 Jan;32(1):71–84. doi: 10.1101/gr.275939.121 (PMC8744676; doi:10.1101/gr.275939.121)
Supplement: Supplemental Material [file supp_gr.275939.121_Supplemental_Figures_.pdf]

SUPPLEMENTAL FIGURES

Meta-analysis of human and mouse ALS astrocytes reveals multiomic signatures of inflammatory reactive states

Oliver J. Ziff[1,2,3 #], Benjamin E. Clarke[1,2 #], Doaa M. Taha[1,2,4 #], Hamish Crerar[1,2],

*Nicholas M. Luscombe[1,5,6], Rickie Patani[1,2,3]**

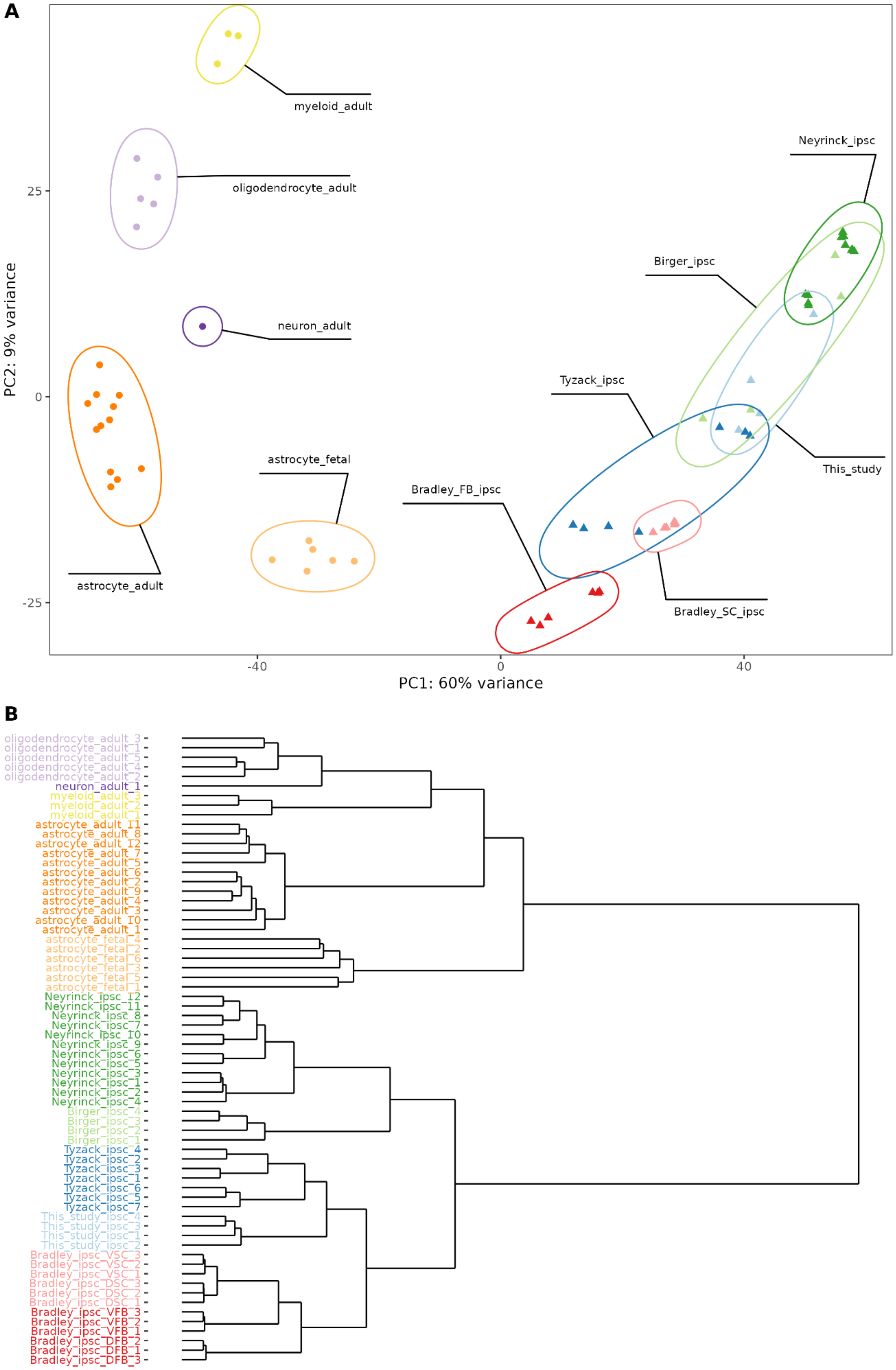
A
myeloid_adult
oligodendrocyte_adult
neuron_adult
astrocyte_adult
astrocyte_fetal
Neyrinck_ipsc
Birger_ipsc
Tyzack_ipsc
This_study
Bradley_FB_ipsc
Bradley_SC_ipsc
PC2: 9% variance
25
0
-25
PC1: 60% variance
-40
0
40
B
oligodendrocyte_adult_3
oligodendrocyte_adult_1
oligodendrocyte_adult_5
oligodendrocyte_adult_4
oligodendrocyte_adult_2
neuron_adult_1
myeloid_adult_3
myeloid_adult_2
myeloid_adult_1
astrocyte_adult_11
astrocyte_adult_8
astrocyte_adult_12
astrocyte_adult_7
astrocyte_adult_5
astrocyte_adult_6
astrocyte_adult_2
astrocyte_adult_9
astrocyte_adult_4
astrocyte_adult_3
astrocyte_adult_10
astrocyte_adult_1
astrocyte_fetal_4
astrocyte_fetal_2
astrocyte_fetal_6
astrocyte_fetal_3
astrocyte_fetal_5
astrocyte_fetal_1
Neyrinck_ipsc_12
Neyrinck_ipsc_11
Neyrinck_ipsc_8
Neyrinck_ipsc_7
Neyrinck_ipsc_10
Neyrinck_ipsc_9
Neyrinck_ipsc_6
Neyrinck_ipsc_5
Neyrinck_ipsc_3
Neyrinck_ipsc_1
Neyrinck_ipsc_2
Neyrinck_ipsc_4
Birger_ipsc_4
Birger_ipsc_3
Birger_ipsc_2
Birger_ipsc_1
Tyzack_ipsc_4
Tyzack_ipsc_2
Tyzack_ipsc_3
Tyzack_ipsc_1
Tyzack_ipsc_6
Tyzack_ipsc_5
Tyzack_ipsc_7
This_study_ipsc_4
This_study_ipsc_3
This_study_ipsc_1
This_study_ipsc_2
Bradley_ipsc_VSC_3
Bradley_ipsc_VSC_2
Bradley_ipsc_VSC_1
Bradley_ipsc_DSC_3
Bradley_ipsc_DSC_2
Bradley_ipsc_DSC_1
Bradley_ipsc_VFB_3
Bradley_ipsc_VFB_2
Bradley_ipsc_VFB_1
Bradley_ipsc_DFB_2
Bradley_ipsc_DFB_1
Bradley_ipsc_DFB_3

Supplementary Figure 1

A: Principal component analysis (PCA) on variance stabilised gene counts plotted by their coordinates along the first two principal components across all human samples. Samples include human biopsy purified cell type samples from Zhang et al. 2016 (Neuron, Myeloid, Oligodendrocyte, adult and fetal Astrocyte) together with hiPSC-astrocytes (This study: *VCP* mutant and control, Tyzack: *SOD1* mutant and control, Birger: *C9orf72* mutant and control, Neyrinck: *FUS* mutant and control and Bradley: regionally specified dorsal (D) and ventral (V) forebrain (FB) and dorsal and ventral spinal cord (SC). Samples are labeled and coloured by their dataset and shape according to source tissue.
B: Dendrogram showing unsupervised hierarchical clustering of variance stabilised gene counts across the same samples.

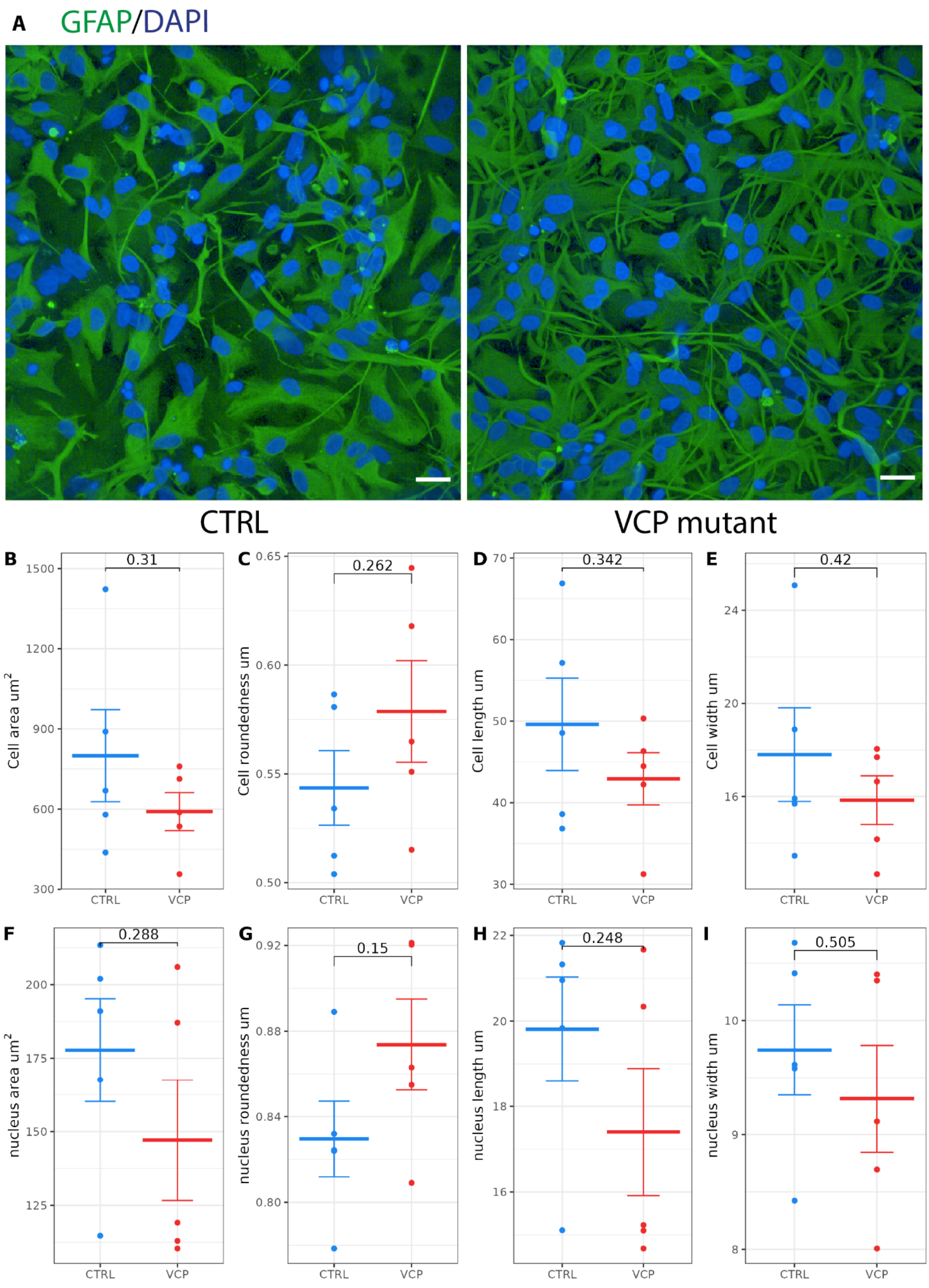

A
GFAP/DAPI
CTRL
VCP mutant
B
0.31
Cell area um2
CTRL
VCP
C
0.262
Cell roundedness um
D
0.342
Cell length um
E
0.42
Cell width um
F
0.288
nucleus area um2
G
0.15
nucleus roundedness um
H
0.248
nucleus length um
I
0.505
nucleus width um

Supplementary Figure 2

A: Representative immunolabeling images of control (left) and *VCP* mutant (right) astrocytes staining for GFAP (green). Nuclei were counterstained by DAPI (blue). Scale bar, 20um.
B-E: Quantification of morphological parameters of whole cell astrocytes in controls (blue, n = 5) and *VCP* mutants (red, n=5).
F-I: Quantification of morphological parameters of astrocyte nucleus in controls (blue, n = 5) and *VCP* mutants (red, n = 5). Results plotted are the mean of 2 technical repeats from 2 experimental repeats. 2-3 biological lines were used in both control and *VCP* mutant for each experimental repeat. P values are reported from the t-test.

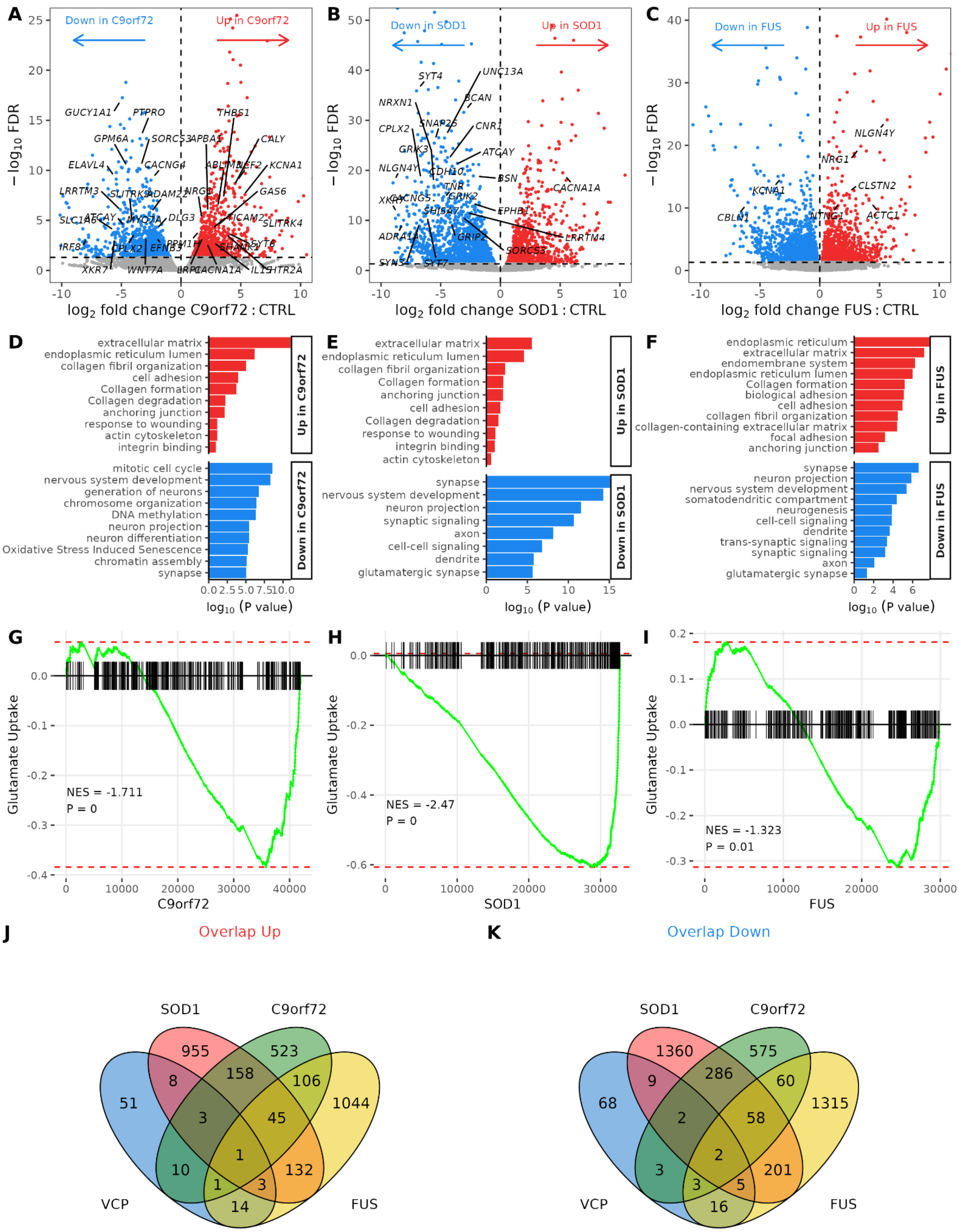

Supplementary Figure 3

A-C: Volcano plots showing $\log_2$ fold change in differential gene expression from RNA sequencing for ALS mutant *C9orf72* (A), *SOD1* (B) and *FUS* (C) versus control astrocytes. Genes with significantly

(FDR < 0.05) increased expression are shown in red and those decreased in expression are shown in blue.

D-F: Gene ontology terms enriched in up (red) and downregulated (blue) differentially expressed genes in ALS mutant *C9orf72* (D), *SOD1* (E) and *FUS* (F) vs control astrocytes.

G-I: Gene set enrichment analyses (GSEA) plots for glutamate uptake in *C9orf72* (G), *SOD1* (H), *FUS* (I).

J-K: Venn diagrams showing overlapping differentially expressed genes between *VCP* (blue), *SOD1* (red), *C9orf72* (green) and *FUS* (yellow) for upregulated (J) and downregulated (K) genes. See Table S3 for details of overlapping genes.

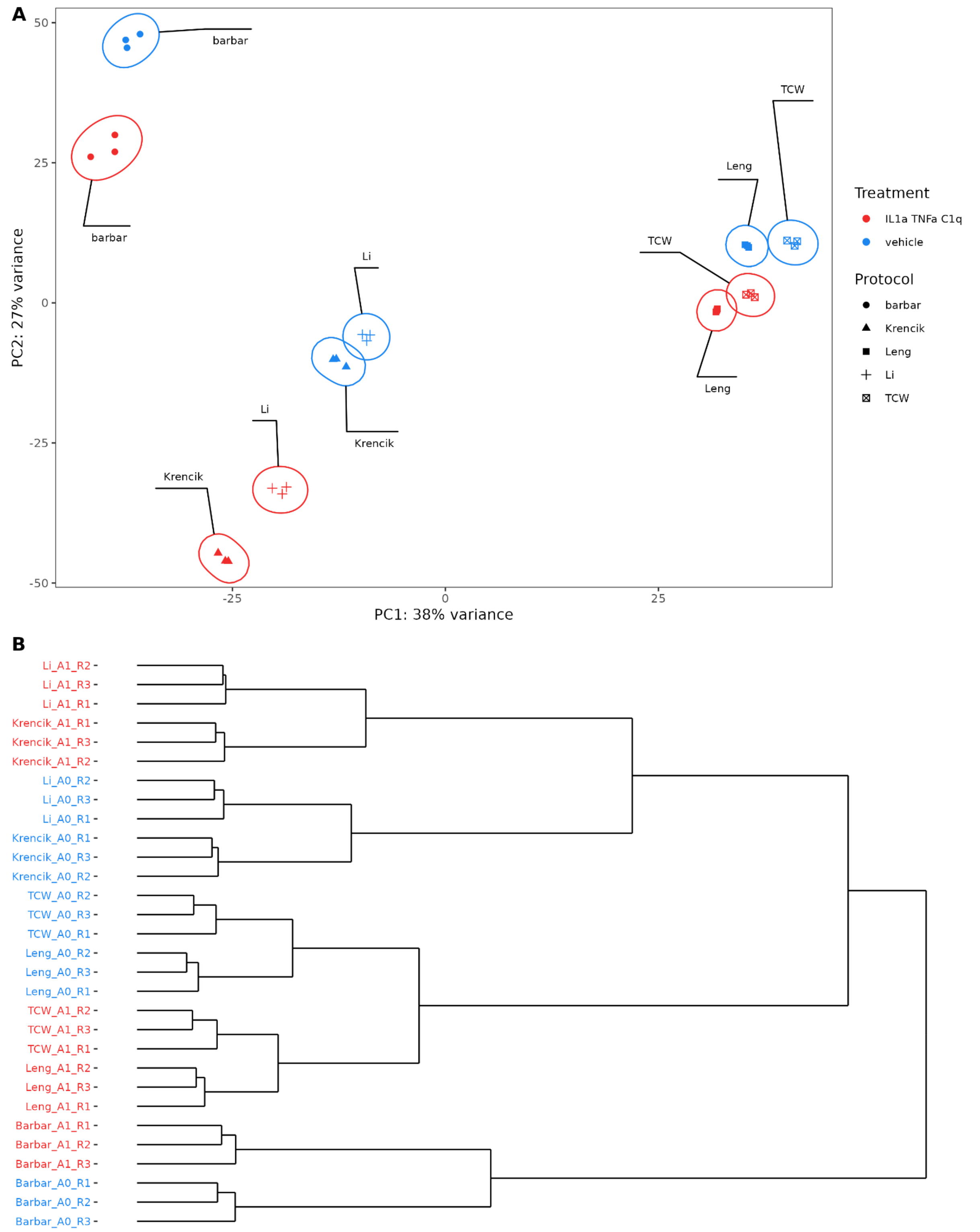

Supplementary Figure 4

A: Principal component analysis (PCA) on variance stabilised gene counts plotted by their coordinates along the first two principal components across TNF, IL1A, C1q and vehicle treated hiPSC derived astrocyte samples.

B: Dendrogram showing unsupervised hierarchical clustering of variance stabilised gene counts across the same samples.

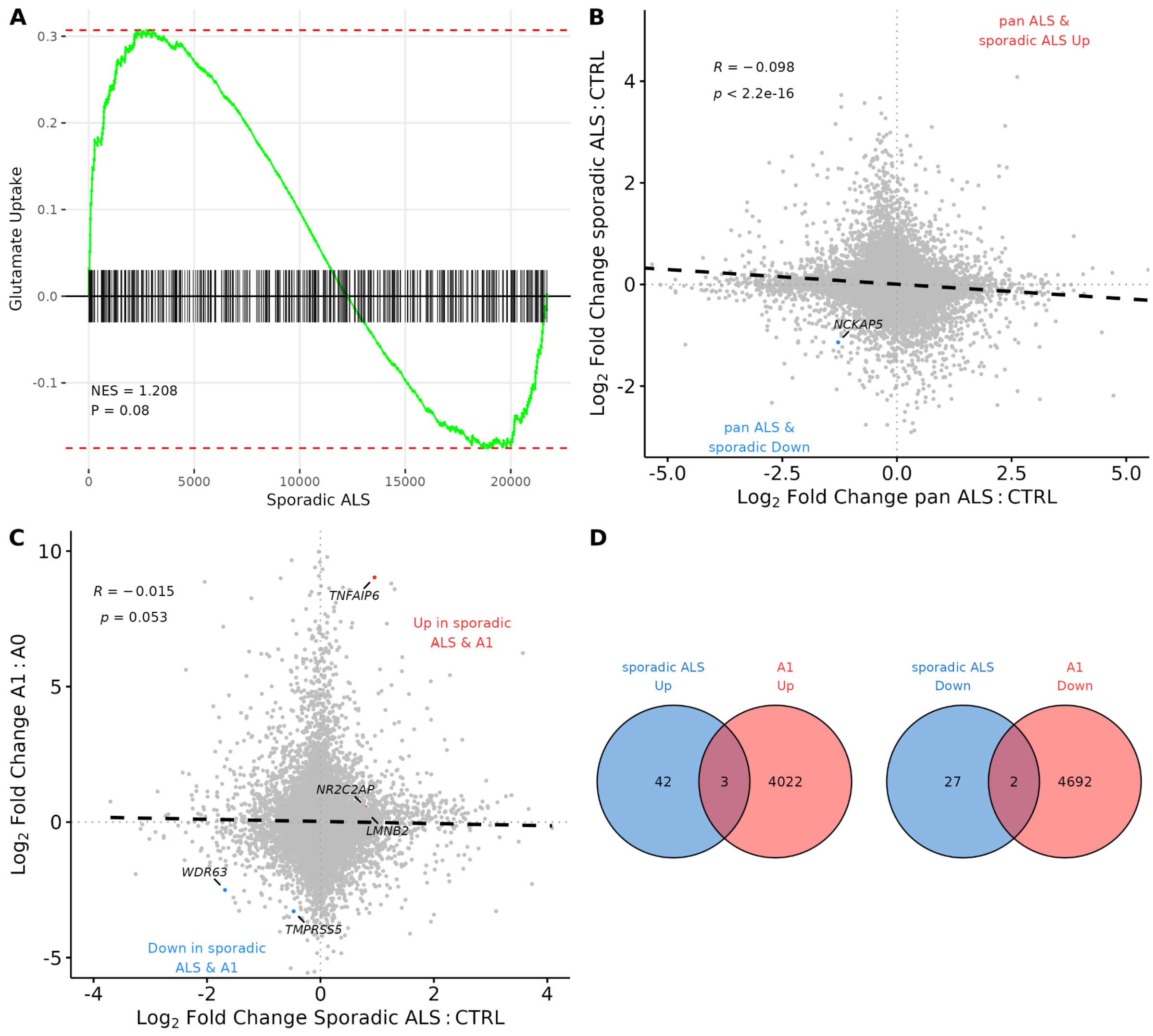

Supplementary Figure 5

A: Gene set enrichment analysis of glutamate uptake in sporadic ALS astrocytes

B: Scatterplot of meta-analysis panALS vs control (x-axis) against $\log_2$ fold change in sporadic ALS vs control microarray.

C: Scatterplot of sporadic ALS vs control (x-axis) against $\log_2$ fold change in hiPSC A1 vs A0

D: Venn overlap of differentially expressed genes in sporadic ALS (blue) and A1 (red) hiPSC astrocytes.

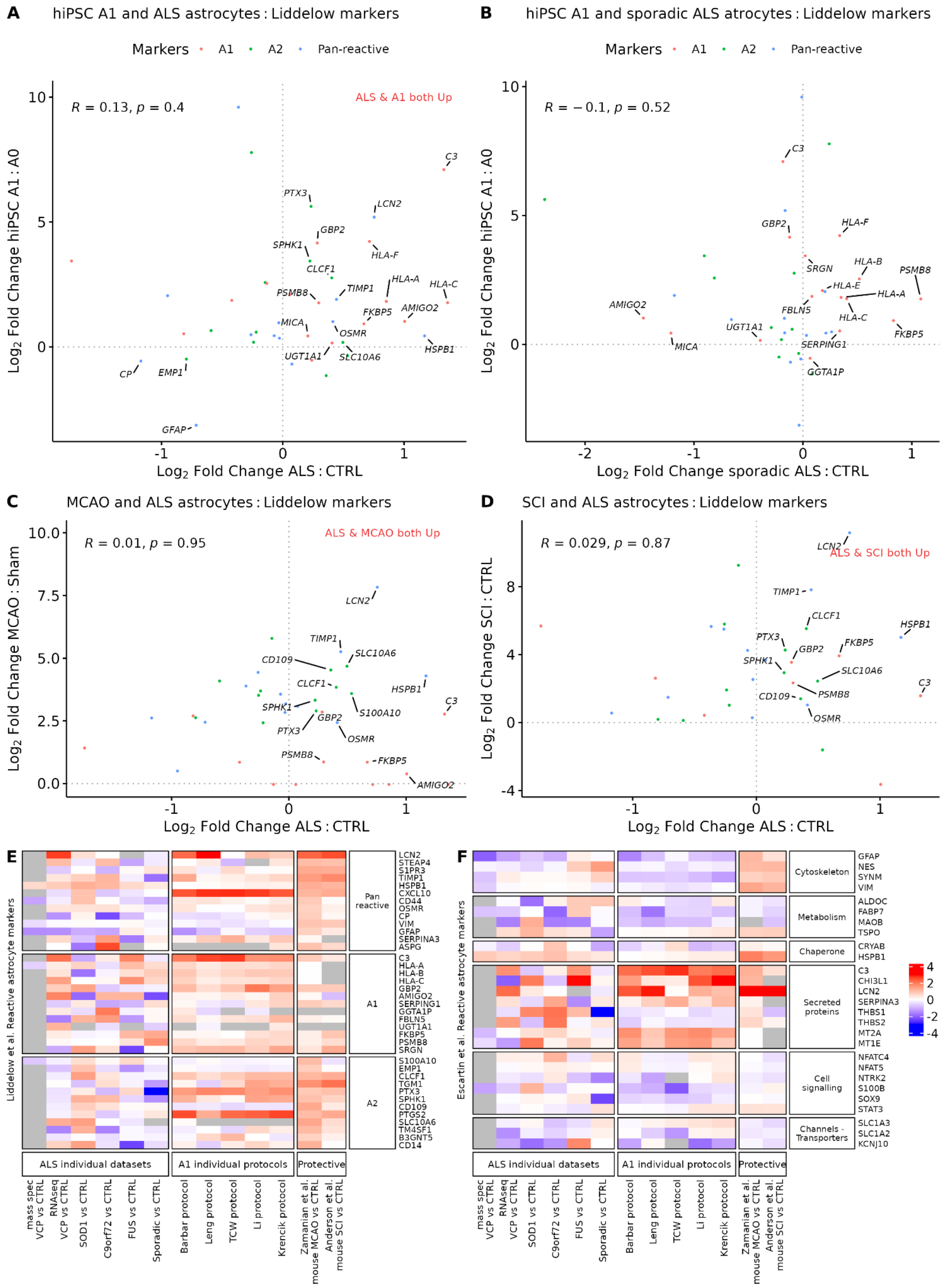

Supplementary Figure 6

A-D: Scatterplots of (Liddelow et al. 2017) astrocyte reactivity markers showing $\log_2$ fold change in hiPSC A1 vs A0 (y-axis) against (A) pan ALS vs control, (B) sporadic ALS vs control (x-axis) and pan ALS vs control (x-axis) against in (C) MCAO vs Sham, (D) SCI vs control (y-axis).

E-F: Heatmaps of (Liddelow et al. 2017) pan-reactive, A1 and A2 specific astrocyte reactivity markers (E) and (Escartin et al. 2021) markers of astrocyte reactivity (F; rows) in the hiPSC ALS astrocyte individual datasets, hiPSC A1 protocols (TNF, IL1A, C1q vs vehicle) and protective astrocyte datasets (columns). Colour intensity represents the scaled differential gene expression $\log_2$ fold changes in each dataset.

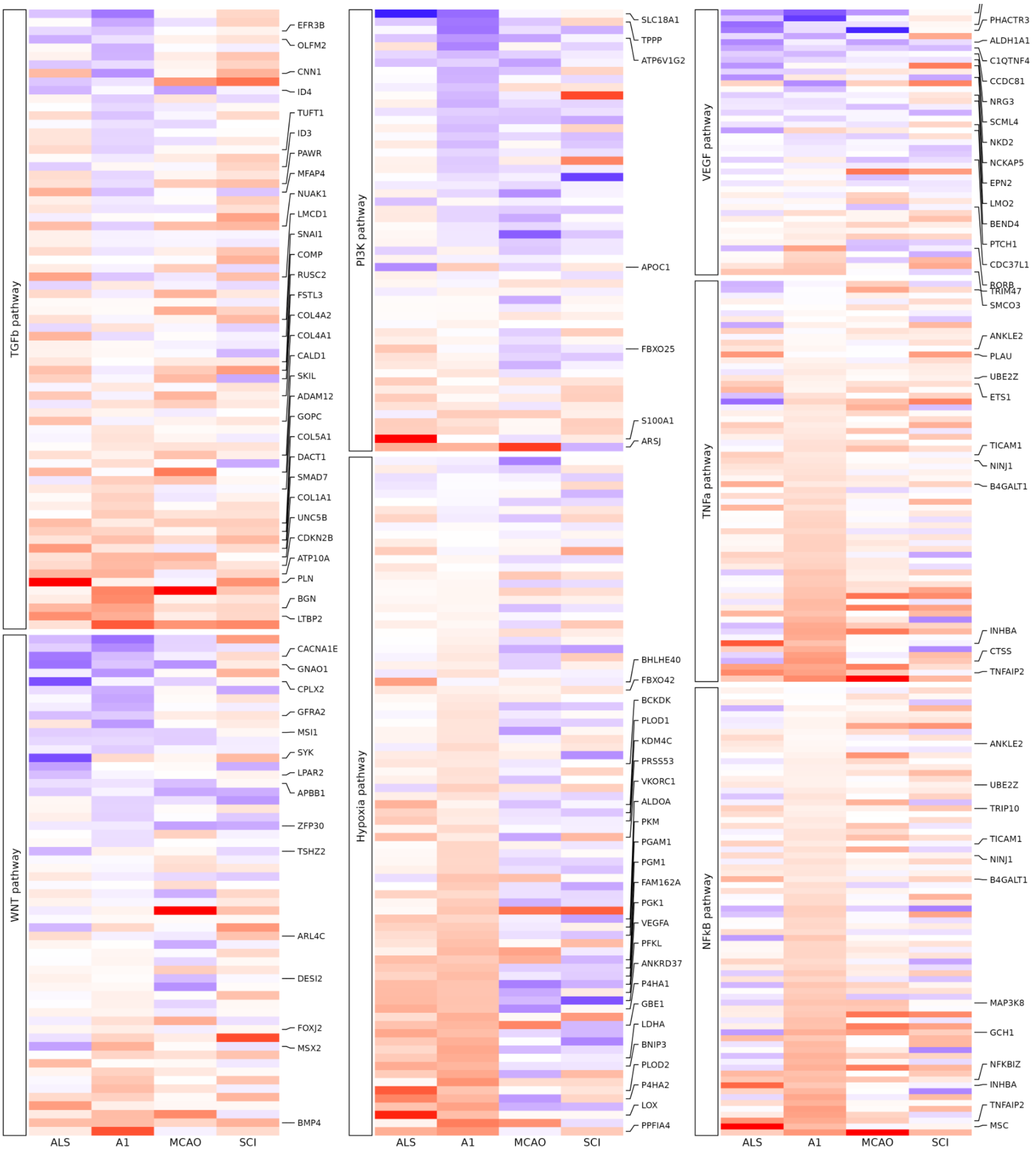

Supplementary Figure 7

PROGENy signaling pathway responsive genes for the top 6 pathways upregulated in pan-ALS vs control. Each heatmap shows the $\log_2$ fold change in (left to right columns) pan-ALS vs control, hiPSC A1 vs A0, MCAO vs sham, SCI vs control. Genes changing significantly in ALS (FDR < 0.05) are labelled.

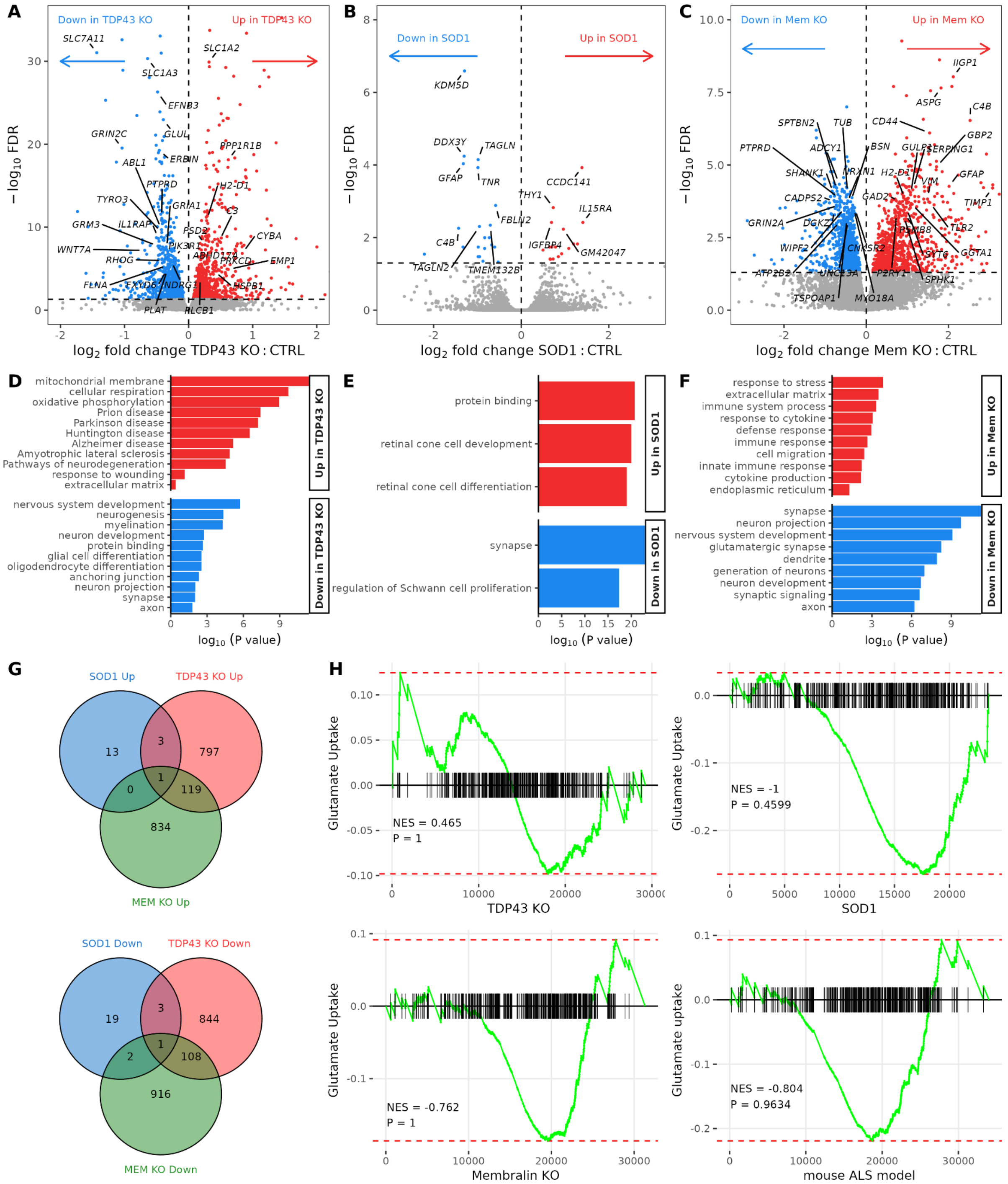

Supplementary Figure 8

A-C: Volcano plots showing $\log_2$ fold change in differential gene expression from RNA sequencing for ALS mutant (A) Tardbp knockout, (B) Sod1^{G93A} and (C) membralin knockout versus control mouse astrocytes. Genes with significantly (FDR < 0.05) increased expression are shown in red and those

decreased in expression are shown in blue.

D-F: Gene ontology terms enriched in up (red) and downregulated (blue) differentially expressed genes in ALS mutant (D) Tardbp knockout , (E) Sod1^{G93A} and (F) membralin knockout vs control astrocytes.

G: Venn diagrams showing overlapping differentially expressed genes between Sod1^{G93A} (blue), Tardbp knockout (red), membralin knockout (green) for upregulated (top) and downregulated (bottom) genes.

H: Gene set enrichment analyses (GSEA) plots for glutamate uptake in Tardbp KO (top left), Sod1^{G93A} (top right), membralin KO (bottom left) and mouse ALS meta-analysis (bottom right).

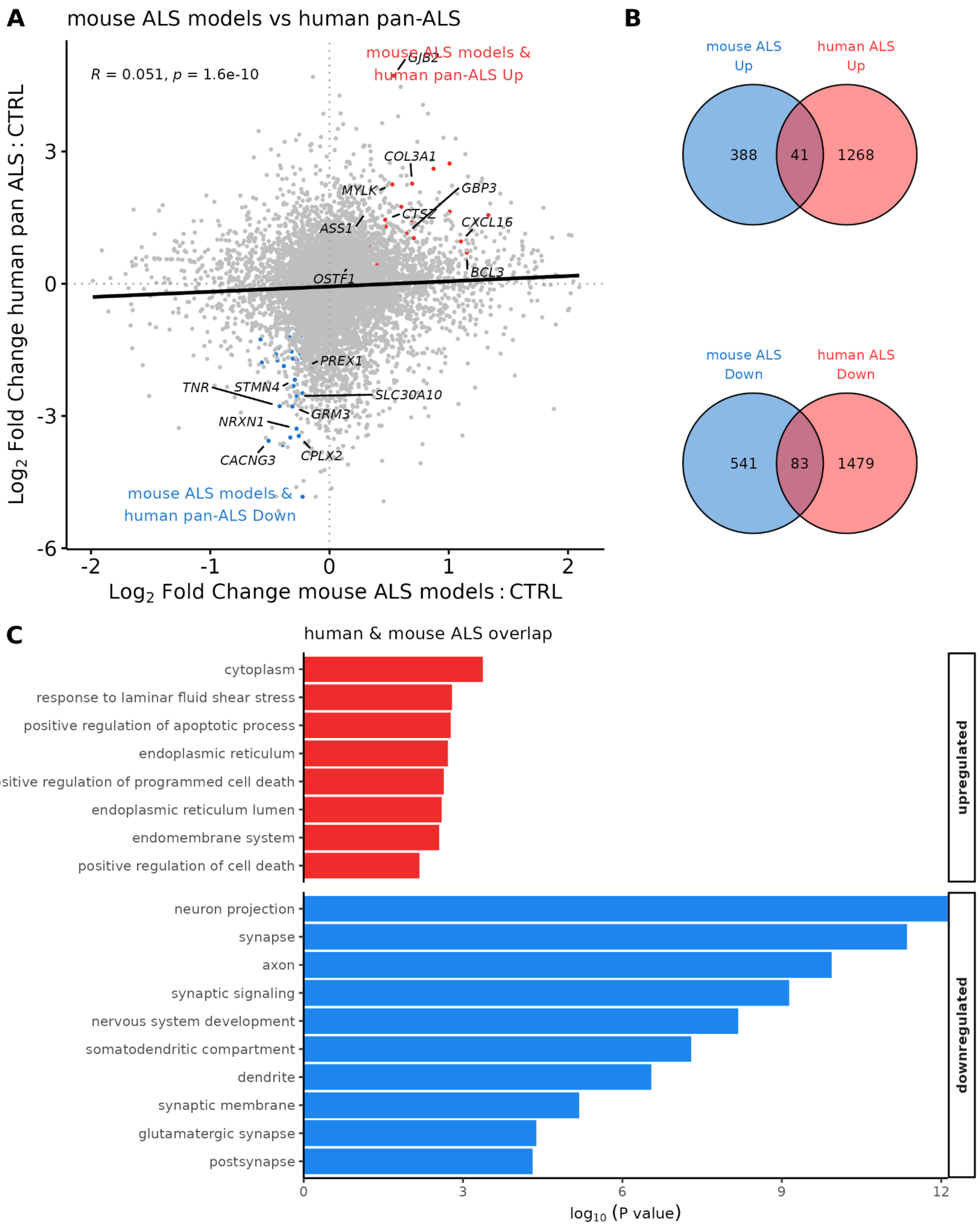

Supplementary Figure 9

A: Scatterplot of human and mouse meta-analyses: log_2 fold change in human ALS vs control (y-axis) against log_2 fold change in mouse ALS models vs control (y-axis). Genes coloured red are upregulated in both meta-analyses whilst blue genes are downregulated in both.

B: Venn overlap of differentially expressed genes in the human ALS meta-analysis (red) and mouse ALS models meta-analysis (blue) for upregulated (top) and downregulated (bottom) genes.

C: Bar graph showing curated overrepresented functional categories (FDR < 0.05) by gene ontology analysis of genes co-upregulated (red) and co-downregulated (blue) between human pan-ALS and mouse ALS model meta-analyses.

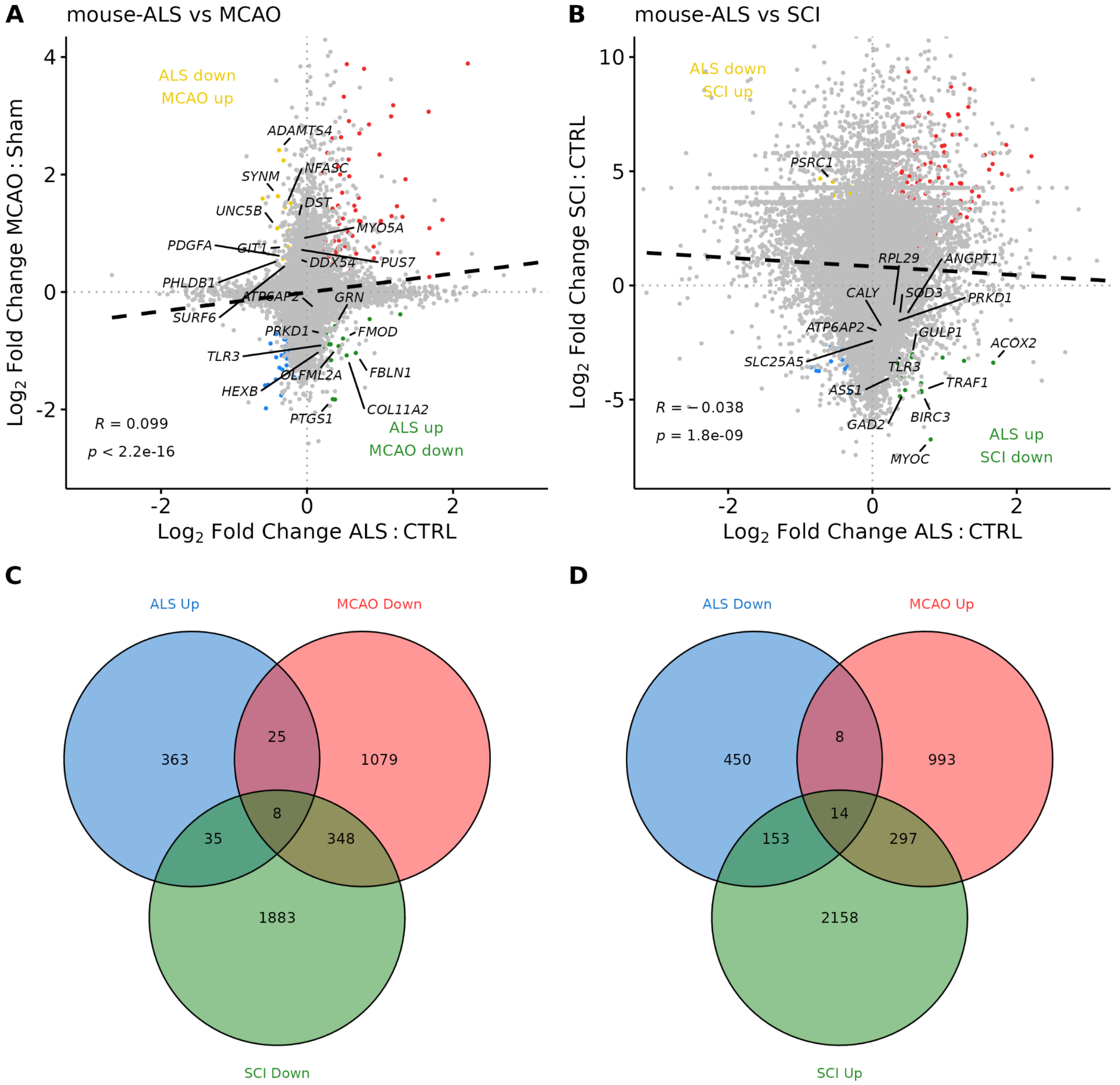

Supplementary Figure 10

A-B: Scatterplots of $\log_2$ fold changes in gene expression in mouse ALS vs control astrocytes (x-axis) against (A) MCAO vs Sham astrocytes and (B) SCI vs control astrocytes (y-axis). Black dashed line indicates linear regression correlation (Pearson correlation MCAO R = +0.1, SCI R = -0.04). Overlapping differentially expressed genes are coloured red (upregulated) and blue (downregulated). Genes increased in ALS but decreased in the protective astrocytes are shown in green, whilst genes decreased in ALS and increased in protective astrocytes are yellow.

C-D: Venn diagram depicting the number of differentially expressed genes (FDR < 0.05) increased

(up) in ALS but decreased (down) in the protective astrocytes (C) and genes decreased in ALS but increased in protective astrocytes (D).

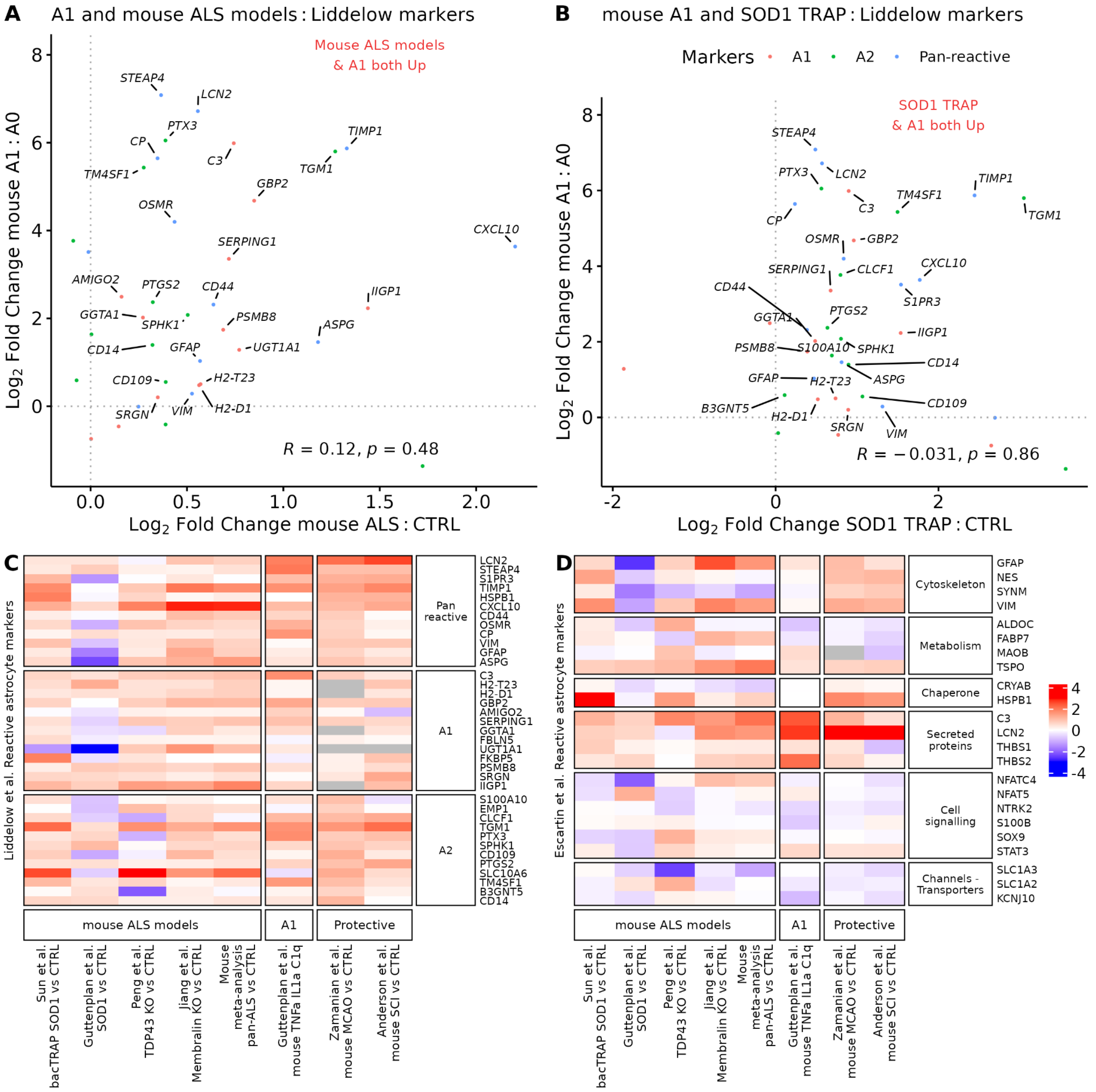

Supplementary Figure 11

A-B: Scatterplots of (Liddelow et al. 2017) astrocyte reactivity markers showing log2 fold change in (A) mouse ALS models vs control and (B) Sod1 TRAP mouse vs control (x-axis) against log2 fold change in mouse A1 vs A0 (y-axis).

C-D: Heatmaps of (Liddelow et al. 2017) pan-reactive, A1 and A2 specific astrocyte reactivity markers (C) and (Escartin et al. 2021) markers of astrocyte reactivity (D; rows) in the mouse ALS models individual datasets (left columns) and meta-analysis and A1 and protective datasets (columns). Colour intensity represents the scaled differential gene expression log2 fold changes in ALS vs control; A1 vs A0 astrocytes.

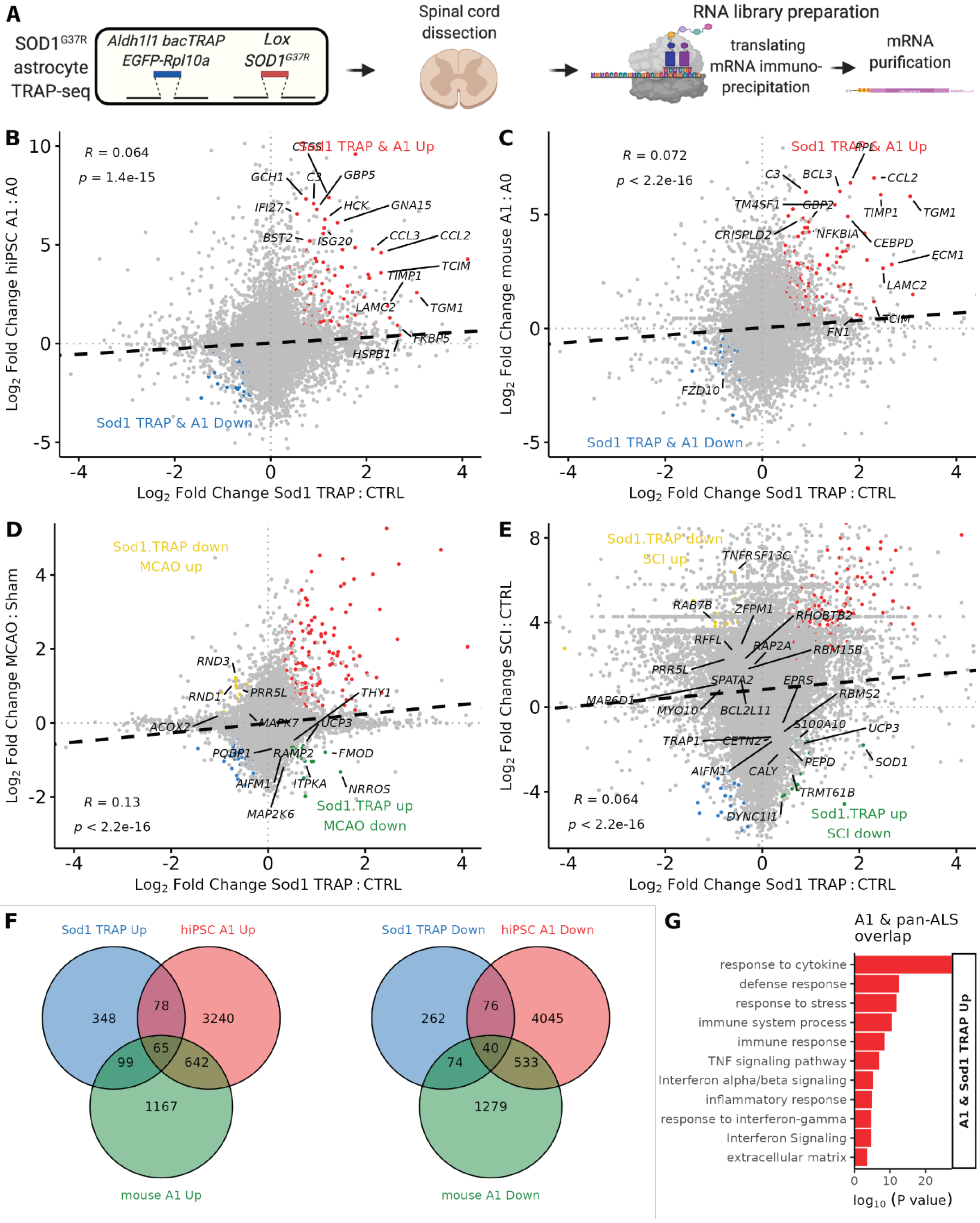

A
SOD1G37R astrocyte TRAP-seq
Aldh1l1 bacTRAP EGFP-Rpl10a
Lox SOD1G37R
Spinal cord dissection
RNA library preparation
translating mRNA immuno-precipitation
mRNA purification
B
Sod1 TRAP & A1 Up
Sod1 TRAP & A1 Down
R = 0.064
p = 1.4e-15
Log2 Fold Change hiPSC A1 : A0
Log2 Fold Change Sod1 TRAP : CTRL
C
Sod1 TRAP & A1 Up
Sod1 TRAP & A1 Down
R = 0.072
p < 2.2e-16
Log2 Fold Change mouse A1 : A0
Log2 Fold Change Sod1 TRAP : CTRL
D
Sod1.TRAP down MCAO up
Sod1.TRAP up MCAO down
R = 0.13
p < 2.2e-16
Log2 Fold Change MCAO : Sham
Log2 Fold Change Sod1 TRAP : CTRL
E
Sod1.TRAP down SCI up
Sod1.TRAP up SCI down
R = 0.064
p < 2.2e-16
Log2 Fold Change SCI : CTRL
Log2 Fold Change Sod1 TRAP : CTRL
F
Sod1 TRAP Up
hiPSC A1 Up
mouse A1 Up
348
78
3240
65
99
642
1167
Sod1 TRAP Down
hiPSC A1 Down
mouse A1 Down
262
76
4045
40
74
533
1279
G
A1 & pan-ALS overlap
response to cytokine
defense response
response to stress
immune system process
immune response
TNF signaling pathway
Interferon alpha/beta signaling
inflammatory response
response to interferon-gamma
Interferon Signaling
extracellular matrix
A1 & Sod1 TRAP Up
log10 (P value)

Supplementary Figure 12

A: Schematic depicting mouse in vivo (top row) ALS Sod1^{G37R} mutant mouse with astrocyte-specific (Aldh1l1) bacTRAP reporter, which expresses the EGFP-tagged ribosome protein (Rpl10a) within astrocytes. Polyribosome-associated mRNAs undergoing translation are then isolated by EGFP immunoprecipitation before mRNA purification and RNA translatome sequencing. B-E: Scatterplots of $\log_2$ fold changes in gene expression in astrocyte-specific bacTRAP mouse Sod1 mutant vs control (x-axis) against $\log_2$ fold changes in (B) hiPSC A1 vs A0 astrocytes, (C) mouse A1 vs A0 astrocytes, (D) middle cerebral artery occlusion (MCAO) vs sham, and (E) spinal cord injury (SCI) vs control (y-axis) against. Black dotted line indicates linear regression correlation (Pearson correlation R reported in each plot). Overlapping differentially expressed genes are coloured red (upregulated, top right quadrant) and blue (downregulated, bottom left quadrant).

F: Venn diagrams depicting the overlap of upregulated (left) and downregulated (right) differentially expressed genes between bacTRAP Sod1 (blue), hiPSC A1 (red) and mouse A1 (green) astrocytes.

G: Bar graph showing curated overrepresented functional categories (FDR < 0.05) by gene ontology analysis of 154 genes Sod1 TRAP, hiPSC & mouse A1 co-upregulated (red). There were no relevant terms enriched amongst the 102 co-downregulated (blue) genes.
